# Lysosome triggered near-infrared fluorescence imaging of cellular trafficking processes in real time

**DOI:** 10.1038/ncomms10855

**Published:** 2016-03-01

**Authors:** Marco Grossi, Marina Morgunova, Shane Cheung, Dimitri Scholz, Emer Conroy, Marta Terrile, Angela Panarella, Jeremy C. Simpson, William M. Gallagher, Donal F. O'Shea

**Affiliations:** 1 Department of Pharmaceutical and Medicinal Chemistry, Royal College of Surgeons in Ireland, 123 St Stephen's Green, Dublin 2, Ireland; 2 School of Chemistry and Chemical Biology, Conway Institute, University College Dublin, Belfield, Dublin 4, Ireland; 3 School of Biomolecular and Biomedical Science, Conway Institute of Biomolecular and Biomedical Research, University College Dublin, Belfield, Dublin 4, Ireland; 4 School of Biology and Environmental Science, Conway Institute of Biomolecular and Biomedical Research, University College Dublin, Belfield, Dublin 4, Ireland

## Abstract

Bioresponsive NIR-fluorophores offer the possibility for continual visualization of dynamic cellular processes with added potential for direct translation to *in vivo* imaging. Here we show the design, synthesis and lysosome-responsive emission properties of a new NIR fluorophore. The NIR fluorescent probe design differs from typical amine functionalized lysosomotropic stains with off/on fluorescence switching controlled by a reversible phenol/phenolate interconversion. Emission from the probe is shown to be highly selective for the lysosomes in co-imaging experiments using a HeLa cell line expressing the lysosomal-associated membrane protein 1 fused to green fluorescent protein. The responsive probe is capable of real-time continuous imaging of fundamental cellular processes such as endocytosis, lysosomal trafficking and efflux in 3D and 4D. The advantage of the NIR emission allows for direct translation to *in vivo* tumour imaging, which is successfully demonstrated using an MDA-MB-231 subcutaneous tumour model. This bioresponsive NIR fluorophore offers significant potential for use in live cellular and *in vivo* imaging, for which currently there is a deficit of suitable molecular fluorescent tools.

## Abstract (web summary)

Real time cellular fluorescence imaging requires a probe that displays high degrees of localisation, low toxicity and good photostability. Here, the authors report a near infrared fluorophore that displays pH-sensitive fluorescence based on phenol/phenolate interconversion, showing real time imaging of cellular processes.

## Graphical abstract

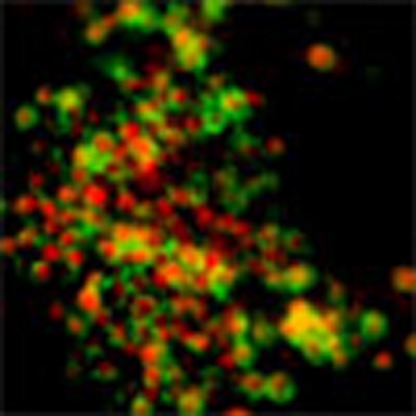

Ehrlich's use of synthetic dyes as a means of staining biological samples can be viewed as one of the foundation stones of modern scientific research. A century later, the use of fluorescence imaging as a technique to visualize specific regions of live cellular^1^,^2^,^3^,^4^ or whole organisms^5^,^6^ is often central to research programmes, with clinical applications such as fluorescence-guided surgery now emerging^7^,^9^,^10^,^11^.

The major shortcomings of fluorescence imaging using molecular fluorophores are interference from nonspecific background fluorescence outside the region of interest (ROI), insufficient photostability and cytotoxicity. Poor ROI selectivity necessitates a time delay to allow background fluorophore clearance and/or a washing procedure between fluorophore administration and image acquisition. This can limit imaging to fixed cells or static snapshots, without the possibility of continuous data acquisition throughout the experiment. An innovative approach to enhance target-to-background signal ratio is to exploit a mechanism of selective fluorescence quenching in the background areas, while establishing the emitting potential of the fluorophore only in the ROI^12^,^13^. Continuous recording of dynamic cellular events in real time may become feasible if the on/off fluorescence switching is reversible.

Developing a responsive fluorophore suitable for real-time live-cell imaging poses a series of challenges. Stringent criteria are required, such as near-perfect response selectivity, exceptional photostability and low dark and light toxicities. Obtaining selective fluorescence responses for intracellular analytes is not trivial, as analyte selectivity observed in a controlled homogeneous environment of a cuvette does not necessarily translate to far more complex *in vitro* or *in vivo* settings. Continuous live-cell imaging places very high demands on photostability of the fluorophore, as the same cell(s) are repeatedly imaged over time. Fluorophore dark toxicity must be low, so that cell viability is not compromised and normal cellular processes are unperturbed. To minimize light-induced toxicity, it is preferable to use low-energy wavelengths in the near-infrared (NIR) spectral region (*λ*=700–900 nm). For *in vivo* imaging, the use of NIR fluorophores is essential. This spectral region is required for effective light transmission through body tissue, as there are reduced levels of absorption and scattering at these longer wavelengths and less intrinsic autofluorescence. In addition, if on/off NIR fluorescence switching could be accomplished *in vivo*, then similar imaging advantages could be gained as for *in*-*vitro* cell imaging.

Currently, there is a small yet growing selection of NIR fluorophore classes but they often suffer from insufficient photostability and lack emission wavelengths above 700 nm^14^. Our recent research focus led to the development of BF_2_-chelated azadipyrromethene class **1** (Fig. 1)^15^,^16^,^17^,^18^. This class is relatively straightforward to synthesize, amenable to structural elaboration and exhibits excellent photophysical properties. For example, the derivative **1** (R=Ph) has an absorption/emission *λ*_max_ at 696 and 727 nm in aqueous solutions, high fluorescence quantum yields (0.3–0.4) and excellent photostability^17^. Yet, in spite of recent progress, a significant need remains for new, more sophisticated intracellularly responsive molecular NIR fluorophores, which can be used to visualize dynamic cellular processes in real time with the potential for *in vivo* translation.

The goal of our current work was to develop an NIR fluorophore capable of a lysosomal-induced off-to-on fluorescence response, thereby permitting real-time imaging of cellular uptake, trafficking and efflux without perturbing function^19^. Endocytosis, the process through which cells internalize biomolecules, is common to all cells and represents a crucial area of research interest due to the numerous associated biological processes^20^,^21^. The participating organelles at each stage in the endocytosis pathway maintain a unique intravesicular/localized pH, to provide appropriate conditions for specific biochemical processes. Although the extracellular and cytosolic regions are at pH ∼7.2, the lysosomes are significantly more acidic. Along the endocytic pathway, the pH lowers from ∼6.3 in early endosomes through ∼5.5 in late endosomes, down to ∼4.5 in lysosomes (Fig. 2)^22^. As such, a difference of almost three orders of magnitude in proton concentration exists between the lysosome interior and the outside of a cell, which is sufficient to establish a selective trigger for fluorescence switching^23^,^24^,^25^,^26^. However, a major additional response selectivity challenge still remains, in that pH-responsive molecular fluorophores can also be responsive to micro-environmental polarity, which can compromise their use in cellular experiments (*vide infra*).

Our novel lysosomal responsive probe design is illustrated in Fig. 1 in which functionalization of the fluorophore core (orange box) with an *ortho*-nitro phenolic group was chosen to impart the pH-responsive feature of the probe. It would be expected that the electron withdrawing *o*-nitro group would result in the ionized phenolate dominating at pH 7.2, resulting in fluorescence quenching due to a non-emissive intramolecular charge transfer excited state (Fig. 1, grey box). Following cellular uptake via endocytosis and compartmentalization into acidic organelles such as lysosomes, protonation would occur giving the neutral phenol species and the NIR emission signal would be established (Fig. 1, red box). This approach is a significant departure from other lysosomal stains, which rely on an amine protonation to form a positively charged ammonium salt to concentrate the fluorophore in the acidic compartments^19^,^22^. An important additional design feature includes a covalently linked polyethylene glycol (PEG) polymer to provide aqueous solubility and promote cellular uptake via endocytic pathways (Fig. 1, blue box)^27^.

## Results

### Synthesis and photophysics

**Figure 1:**
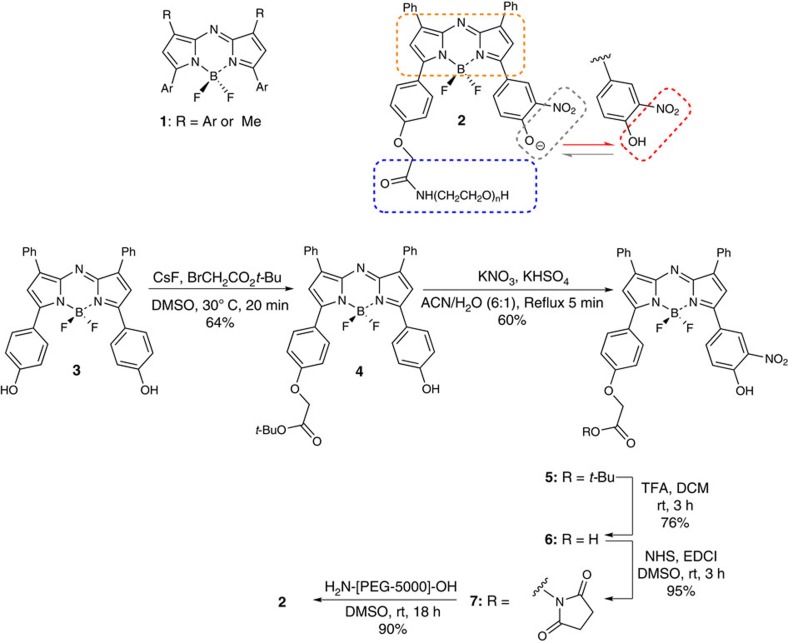
BF_2_-azadipyrromethene NIR fluorophores. General structure of BF_2_-azadipyrromethenes **1**. Design and synthesis of lysosomal responsive BF_2_-azadipyrromethene NIR fluorophore **2**.

The starting point of the synthesis was the previously reported BF_2_-chelated bis-phenol azadipyrromethene **3**, accessible in three synthetic steps from 1-(4-hydroxyphenyl)-3-phenylpropenone (Fig. 1)^28^. Mono-alkylation of **3** was achieved to produce **4** by reaction with *t*-butyl bromoacetate and CsF in dimethylsulfoxide (DMSO) at 30 °C. After isolation, compound **4** was then subjected to *ortho*-nitration of the remaining phenol ring with KHSO_4_/KNO_3_ to provide **5**. Next, hydrolysis of the *t*-butyl ester of **5** with trifluoroacetic acid (TFA) gave the carboxylic acid **6**, which was converted into its activated ester **7** by reaction with *N*-hydroxysuccinimide and *N*-(3-dimethylaminopropyl)-*N*′-ethylcarbodiimide in DMSO. Formation of the activated ester was monitored by ^1^H NMR via the diagnostic CH_2_ peaks at 5.48 for **7** and 4.83 p.p.m. for carboxylic acid **6**, which showed complete conversion within 2 h (Supplementary Fig. 1). Conjugation of **7** in DMSO with a terminal amine functionalized PEG polymer (average molecular weight of 4,900) was effective, with the final fluorophore **2** obtained in high yield (Fig. 1). Matrix-assisted laser desorption/ionization–time of flight (MALDI–TOF) analysis of **2** showed the expected molecular weight centred at 5,410 Da, indicating that the covalent linkage was effective. Furthermore, ^1^H NMR was consistent with the product structure and analytical high-performance liquid chromatography (HPLC) showed a single peak for **2** with retention time differing from that of both the acid **6** and ester **7** (Supplementary Fig. 2).

Comparative absorption and fluorescence emission spectra were recorded for the organic soluble fluorophore **5** in chloroform and aqueous soluble **2** in phenol red-free imaging DMEM medium adjusted to pH 2 (Fig. 3a). Only small differences were observed between the two fluorophores in the differing organic and aqueous media. Encouragingly, probe **2** had fluorescence *λ*_max_ at 707 nm with an absorbance *λ*_max_ at 685 nm. Extinction coefficient and fluorescence quantum yield values for **5** and **2** were similar with polyethylene glycol-substituted **2** having values of 97,000 cm^−1^ M^−1^ and 0.18, respectively (Fig. 3a).

An undesirable feature of some pH-sensitive fluorophores is their strong sensitivity to micro-environmental polarity, which significantly compromises their use in biological settings^29^,^30^,^31^. To test the polarity sensitivity of **2**, its acid/base emission-responsive properties were recorded in toluene, tetrahydrofuran, dimethylformamide and DMSO for both the phenol and phenolate state using 1,8-diazabicyclo[5.4.0]undec-7-ene (DBU) and TFA to cycle between the two (Fig. 3b). A plot of solvent polarity function (Δ*f*)^32^ versus integrated fluorescence intensities in the off states showed highly effective fluorescence quenching as the phenolate irrespective of solvent polarity. A strong fluorescence output was established once protonated to the phenol in all solvents (Fig. 3c). These results predict that the modulation of fluorescence intensity would be selective for pH changes, while remaining unresponsive to differing intracellular micro-environmental polarities, thereby removing the potential for false-positive emissions. An identical study was carried out for fluorophore **5**, giving similar results and indicating that this positive feature is general to the fluorophore class (Supplementary Fig. 3).

As sufficient photostability is an essential property for prolonged live-cell imaging, a comparative study of the photodegradation of **2**, lysotracker red and pH-rhodo red was carried out. DMEM solutions of the three fluorophores were illuminated with light of 620(30) nm for **2** and 540(40) nm for lysotracker red and pH-rhodo red for 2 h, and their fluorescence intensity monitored. Encouragingly, no photobleaching for **2** was observed, whereas both other fluorophores were ∼80% degraded within that time frame (Fig. 3d). Comparison of their stabilities in HeLa Kyoto cells using illumination from a solid-state light emitting diode (LED) light source was also examined. Cells stained with **2**, lysotracker red or pH-rhodo red were constantly illuminated with LED power set to a maximum, to promote a fast rate of photobleaching. The same excitation filters used for imaging (640(14) nm for **2** and 563(9) nm for lysotracker red and pH-rhodo red) were used, allowing images to be acquired at various time intervals. Graphing the average cell fluorescence intensity versus time showed that **2** was the most photostable with 50% loss of signal in 94 s and lysotracker red being the least photostable with 50% of signal loss in just 6 s (Fig. 3e, Supplementary Fig. 4 and Supplementary Movies 1 and 2). The behaviour of pH-rhodo red was more complex, as its intensity first significantly increased throughout the cell followed by photobleaching (Fig. 3e, Supplementary Fig. 4 and Supplementary Movie 3). This response to irradiation is indicative of a photo-conversion occurring for pH-rhodo red but further studies would be required to fully establish the cause for this. Comparison of these results highlights the distinct advantage of **2** for prolonged live-cell imaging in which fluorophore photostability is an essential parameter.

The pH-responsive properties of **2** were investigated in DMEM containing 10% fetal bovine serum (FBS) before its use in imaging studies. Fluorescence output of **2** was negligible at pH 7.4, but became highly fluorescent at acidic pH with its p*K*a determined as 4.0 (Fig. 4a). Cy5.5 light filter parameters of 690/50 nm were applied to the emission bands at pH 7.4, 5.5 and 4.5, and the integrated fluorescence intensity differences determined. At pH 5.5, as found in late endosomes, the fluorescence enhancement factor (FEF) was 6-fold, while it reached a remarkable 21-fold at lysosomal pH of 4.5 (Fig. 4b). Taken together, these results predict that at a cellular level **2** would remain non-fluorescent in the extracellular environment and become highly NIR fluorescent on uptake and localization in the lysosomes (Fig. 4c). The difference in emission intensity at pH 5.5 (late endosomes) and 4.5 (lysosomes) suggests that the increased activation of **2** in lysosomes may be sufficient to allow differentiation between these organelles.

### *In vitro* fixed and real-time live-cell imaging

Before testing the imaging capabilities of **2**, its cytotoxicity in HeLa Kyoto (cervical cancer) and HEK (human embryonic kidney) cell lines was determined. Following a 24-h incubation of cells with **2**, an MTT (3-(4,5-dimethylthiazol-2-yl)-2,5-diphenyltetrazolium bromide) assay was performed and EC_50_ values of 0.43 and 0.44 mM, respectively, were obtained (Supplementary Fig. 5). These values were used as the basis to select 10 μM as the concentration for its use in imaging experiments. To establish the ability of **2** to internalize in cells, HeLa Kyoto and HEK293 cells were incubated with **2** for 2 h, followed by fixation, nuclei staining (Hoechst 33342) and imaging with confocal laser scanning microscopy (CLSM) (Fig. 5). These images show that **2** was internalized in both cell lines within 2 h (Supplementary Movie 4). The fluorescence signal was predominately localized in the perinuclear region as would be expected for a lysosomal staining pattern, indicating that the fluorophore had accumulated and become fluorescent in lysosomes^19^.

To gain further evidence of selective lysosomal staining, an identical experiment was performed using a HeLa cell line stably expressing the lysosomal-associated membrane protein 1 (LAMP1) fused to green fluorescent protein (GFP)^33^. Following incubation, CLSM-imaged cell images showed very high levels of co-compartmentalization of the red (**2**) and green channel (GFP) emissions to the lysosomes (Fig. 6). Examination of selected focal planes clearly showed a circumferential staining pattern (green) of the LAMP1-GFP in the lysosome membrane and red emission of **2** from within the acidic lumen of the organelles (for Z-stack see Supplementary Movie 5). This ability to resolve the lysosome membrane from the interior is approaching the confocal resolution limit, with ∼1 μm being the average diameter of an individual lysosome (for images from an additional independent experiment, see Supplementary Fig. 6). This was achieved due to the high signal-to-noise ratio, as background emission from **2** is not observed, and high red/green contrast with the genetically expressed LAMP1–GFP. Further statistical evidence of co-compartmentalization of the NIR and green emissions was provided by the calculated Manders' coefficients of 0.91 (05) for *M*_NIR_ and 0.95 (05) for *M*_green_ and a Pearson's coefficient of 0.79 (09)^34^. The lower Pearson's coefficient value when compared with Manders' may be attributable to the fact that the NIR and green emissions are co-compartmentalized to the lysosomes but not fully co-localized as the green is in the outer membrane and the NIR from within the internal lumen. Golgi co-staining of HeLa cells with **2** showed no significant co-localization (Supplementary Fig. 7, Method 1).

Two possible explanations for the excellent co-compartmentalization of red and green emissions could be envisaged. Either **2** is physically located exclusively in the lysosomes or it is present in other organelles along the endocytic pathway and is only emissive from the lysosomes due to its lower pH, with the remaining **2** being fluorescent silent. To visualize all intracellular **2** in its fluorescent on state, HeLa cells were incubated with **2** in media at pH 7.4 for 2 h, nuclei stained with 4,6-diamidino-2-phenylindole (DAPI) for 15 min, fixed and imaged using CLSM (Fig. 7a and Supplementary Movie 6). The media containing the fixed cells was then changed for media at pH 4.9 (adjusted using HCl), which on penetrating the cell forced on the fluorescence of all **2** within cells not localized in a sufficiently low pH environment. Re-imaging of the same cells (30 min after media change) with identical microscope settings showed a significantly increased fluorescence with saturation of the field of view within the cells (Fig. 7b and Supplementary Movie 7). The mean corrected total fluorescence from the cells (calculated using ImageJ) showed an FEF of 9.5 on lowering of the pH (see Supplementary Fig. 8 for images from additional independent experiment). This clearly illustrates that fluorescence from the majority of **2** was not switched on by the cells and only **2** localized in the lysosomes was emissive under normal pH conditions. The same field of view was imaged for the third time using adjusted microscope laser power and photomultiplier tube (PMT) voltage and it became clear that the additional fluorescence of **2** was predominantly from other cellular organelles of higher pH (Fig. 7c and Supplementary Movie 8). Unfortunately, the adjustment to lower pH caused a loss of the LAMP1–GFP signal; thus, a comparison of Manders' coefficients was not possible.

To show that fluorophore **2** was internalized in cells via endocytosis and not passive diffusion, HeLa Kyoto cells were incubated at 4 °C for 30 min with **2**. It is known that endocytosis is inhibited at 4 °C, whereas passive diffusion can still occur^35^. Following incubation, cells were imaged, the buffer adjusted to pH 4.9 and re-imaged as described above. In contrast to the result shown in Fig. 7, no fluorescence from **2** was detected before or after changing the buffer to pH 4.9 (Supplementary Fig. 9). From these experiments, it was concluded that **2** was internalized via an energy requiring endocytosis rather than passive diffusion.

For the results outlined above, it was anticipated that cellular uptake of **2** could be continuously imaged in real-time without the need for washing or manipulating cells. The first live-cell imaging experiment involved imaging cells in a single focal plane over a 1.5-h time period. Once in focus, HeLa Kyoto cells were treated with **2** and imaged with an epi-fluorescence live-cell microscope, under optimal conditions of temperature and atmosphere for the cells to remain fully active (37 °C and 5% CO_2_ humidified environment). Images were continuously acquired every 30 s for 90 min and then combined to form a movie (Supplementary Movie 9). Representative time-lapse images after 1, 30, 60 and 90 min (in black/white for clarity) are shown below in Fig. 8a with the 90 min time point in red for comparison (Fig. 8b). The first image acquired at 1 min showed no NIR fluorescence, a signal confirming the effective fluorescence quenching of extracellular **2**. However, over the following 90-min time period, a strong signal arose from point-like organelles as a result of cellular uptake of **2** and transport through the endocytic pathway to the lysosomes (Fig. 8: 60- and 90-min time points). On close inspection, individual lysosomes can be seen emerging into view over the first 30-min time period, following which they increase in intensity and number from 30 to 90 min (Supplementary Movie 9).

The continuous imaging of live cells in the *z*-axis provides the most realistic method for following the progress of biological events over time and is of particular relevance when imaging small mobile organelles. To generate a three-dimensional (3D) representation of the cell in real time, a *z*-stack of 25 focal planes through the cell was acquired every minute^36^. This continual recording of cellular 3D volume over a period of time is termed four-dimensional (4D) imaging as the sample is imaged in the *x*,*y*,*z* and time dimensions, from which a time-lapse video of the 3D cellular volume can be created. Using HeLa cells, a 4D data set of the uptake of **2** over a 60-min time period was acquired followed by deconvolution of the data set to correct for motion of fluorescence objects between focal planes during the 1-min time period required to complete a *z*-stack of the cell. This experiment showed punctuated regions of fluorescence over the 60-min time period starting from a non-fluorescent background at time zero with individual lysosome movement clearly observable (Fig. 9a,b and Supplementary Movie 10). Quantification of the fluorescence increase was measured by selecting two identical volumes of the imaged area, one overlapping with a chosen cell and the other on the extracellular environment, and applying an image analysis algorithm (ImageJ) to measure the total fluorescence intensity within the volumes (Fig. 9c). A comparative plot of both intensities over time shows how the background remained non-fluorescent, whereas intracellular fluorescence intensity increased over time, reaching a plateau at 60 min at which point a dynamic equilibrium was established between extracellular and intracellular **2**.

This ability to 4D image with **2** provides a tool for tracking lysosomal movements within the cell. Lysosomal staining of HeLa Kyoto cells was achieved by incubation with **2** for 1 h following which they were imaged in 3D for 35 min (without medium replacement) using a widefield microscope. Image analysis software was used to tag individual lysosomes as white spheres to facilitate visualization and the movement of these lysosomes was tracked over the 35-min time period (Fig. 10). The path the lysosome takes through the cell is illustrated by a lengthening white tail, which extends from the lysosome as the video progresses (Fig. 10c,d and Supplementary Movie 11)^37^. Tracking of all lysosomes within a cell (or field of view) was also possible and is shown for Fig. 10 in the Supplementary Information (Supplementary Movie 12).

Our final *in vitro* experiments with pH-responsive **2** were aimed at studying the efflux of the fluorophore from the cell. HeLa Kyoto cells were pre-treated with **2** for 2 h as previously described, then the medium was replaced with fluorophore-free DMEM. Cells were incubated at 37 °C and imaged at time points of 1, 15, 30 and 120 min using confocal microscopy (Fig. 11). The overall NIR fluorescence detected after 2 h incubation with **2** in the intracellular environment showed to be again arising from point-like organelles (Fig. 11a). The quantity of fluorescent vesicles decreased within 30 min after medium change, with significant clearance of **2** by 120 min (Fig. 11b). Using ImageJ analysis, lysosome number per cell (or field of view) was determined for the different time points and were observed to steadily decrease from time 0 to 120 min (Fig. 11d). In addition, after 60 min cells were fixed and acidified with buffer of pH 4.9 and only a small, 1.6-fold increase in total fluorescence was observed.

### *In vivo* imaging

A distinct advantage of NIR fluorophores is their ability to directly transfer from *in vitro* to *in vivo* imaging due to transparency of biological tissue at these longer wavelengths. To test *in vivo* performance of **2**, the luciferase-expressing human breast cell line MDA-MB-231-luc-D3H1 was chosen to grow subcutaneous tumours of size 100–200 mm^3^, which permitted NIR fluorescence imaging with confirmatory bioluminescence. The ability of PEG polymers to act as a drug delivery vehicle has been well established, with several PEGylated drugs in clinical use for over 20 years. PEGylation is known to influence pharmacokinetic properties resulting in prolonged blood circulation times. As such, it was anticipated that the PEG-conjugated **2** may have passive tumour-targeting properties leading to some preferential uptake into tumour cells, thereby generating a distinguishable fluorescent signal. Following an intravenous tail vein injection of **2** (2 mg kg^−1^), images were acquired at regular intervals over the course of 24 h. A plot of tumour to background NIR fluorescence showed that the fluorescence signal was low in the beginning in both the background and tumour. Emission from the liver peaked at 1 h and subsided over the following 24 h (Fig. 12d dashed red line). In contrast, the tumour fluorescence intensity reached a maximum at 24 h, allowing for good image discrimination at that time point (Fig. 12d solid red line). Bioluminescence and NIR fluorescence imaging at 24 h confirmed that **2** has indeed been taken up into tumour cells and switched on (Fig. 12a–c). No adverse reactions or animal weight loss were observed during or after imaging. These preliminary *in vivo* results represent a unique example of selective NIR *in vivo* tumour imaging using a pH-responsive fluorochrome.

## Discussion

**Figure 2:**
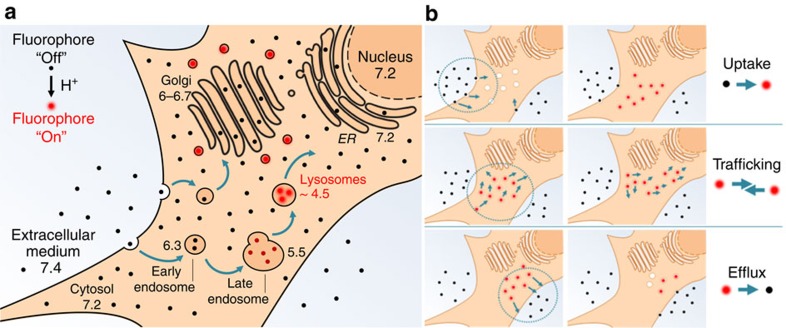
Cellular uptake responsive NIR-fluorophore. (**a**) Simplified endocytosis of a responsive NIR fluorophore. Numbers represent the approximate pH of the corresponding organelles. (**b**) Three observable stages of the path of the pH-responsive fluorophore in the cellular environment: uptake, trafficking and efflux.

**Figure 3:**
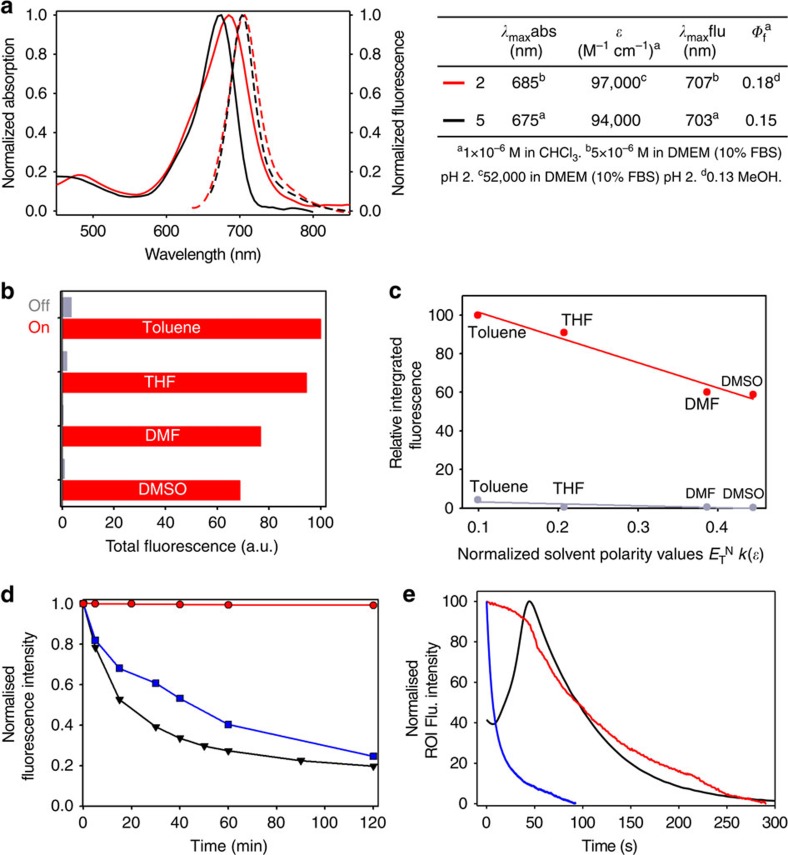
Photophysical properties of NIR-fluorophores. (**a**) Light absorption and emission spectra of compounds **2** and **5**, and their photo-physical parameters. (**b**) Integrated off and on fluorescence states of **2** (5 × 10^−6^ M) in toluene, tetrahydrofuran (THF), dimethylformamide (DMF) and DMSO with TFA (red bars) and DBU (grey bars). (**c**) Plot of relative off and on integrated fluorescence versus solvent polarity values for toluene, THF, DMF and DMSO. (**d**) Comparative photobleaching of 1 × 10^−7^ M DMEM solutions of **2** (red line), lysotracker red (blue line) and pHrodo red (black line) with 150 W fibre optic delivered light 620(30) nm for **2** and 540(40) nm for lysotracker red and pH-rhodo red at 25 °C. (**e**) *In vitro* photobleaching of **2** (red), lysotracker red (blue line) and pHrodo red (black line) with maximum LED power using excitation filter 640(14) nm for **2** and excitation filter 563(9) nm for lysotracker red and pH-rhodo red.

**Figure 4:**
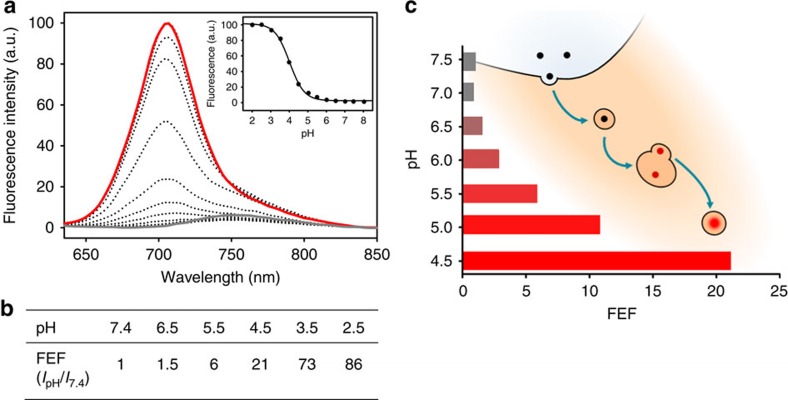
Cellular uptake responsive NIR-fluorescence. (**a**) Emission spectra of **2** (5 × 10^−6^ M) in DMEM (10% FBS) at pH ranging from 8 (grey) to 2 (red). Exc: 625 nm. Inset: fluorescence intensity at *λ*_max_=707 nm versus pH; sigmoidal plot fit resulted in apparent p*K*_a_=4.0. (**b**) Corresponding FEF values from differing pH solutions applying Cy5.5 filter parameters. (**c**) Diagram represents the pH changes and increasing fluorescence intensity along endocytic path towards lysosomes.

Imaging with molecular fluorophores is an indispensable tool for all forms of biological and medical research. Although the full spectrum of colours are available from molecular fluorophores, imaging with lowest energy NIR spectral region offers advantage for prolonged live cellular imaging with the possibility of *in vivo* imaging with the same probe^5^,^37^. Although most fluorescent markers are permanently fluorescent (unless photobleached), huge imaging advantage can be gained if the fluorescence can be modulated from off to on in a reversible bioresponsive manner^14^. With these goals in mind, we have designed a lysosomal-responsive NIR probe with the potential for real-time visualization of their key cellular operations that can be directly translated for use *in vivo* (Figs 1 and 2). NIR probe **2** is synthesized in five synthetic operations from a bisphenolic substituted member of the BF_2_-azadipyrromethene fluorophore class (Fig. 1). An *o*-nitro-substituted phenol group on the probe acts as the fluorescent switch with the nitro group tailoring the emission response to the lumen microenvironment of the lysosomes. Photophysical measurements in DMEM shows that **2** remained fluorescent silent at pH 7.2, yet became highly fluorescent in the pH range that corresponds with the acidic micro-environment of the lysosome (Fig. 4). The non-fluorescent state shows little polarity sensitivity, indicating that it could be used in the more complex intracellular environment without resulting in false-positive emissions. Solution and cellular photobleaching experiments indicated high stability, which is an essential feature for imaging over prolonged time periods that is often lacking in both synthetic and genetically expressed probes (Fig. 3).

To illustrate the potential uses of probe **2**, a series of increasingly complex imaging experiments were undertaken in fixed, live cells and *in vivo*. The high fidelity for the switching on of **2** to the lysosomal lumen is observed when imaging with LAMP1-GFP HeLa cells (Fig. 6). We have exploited these responsive emission properties for 3D and 4D real-time live-cell imaging of several fundamental biological events, such as endocytosis, organelle trafficking and efflux. As **2** has extremely low emission in cell media and with fluorescence activated on cellular internalization, a high background-to-noise ratio is achieved, making the continuous acquisition of data as straightforward as just add **2** to cells and image. It could be anticipated that these techniques would be valuable for many types of cellular experiments involving lysosomal response to stimuli.

It is important to note that the mode of action and the use of existing lysosomotropic stains, which are typically amine-functionalized fluorophores to promote retention in acidic lysosomes on protonation, is significantly different from **2** (refs 38, 39). These lysosomotropic fluorophores are pre-incubated for a period of time (typically 30 min), followed by cell washing to remove excess nonspecific fluorophore, before imaging can be carried out for up to a maximum of 1 h (ref. 40). In contrast, **2** in its fluorescent on state is uncharged, showing little cytotoxic effect over long incubation times and no background fluorescence due to the highly selective switching ‘on' only in the desired lysosomal ROIs. These characteristics have been demonstrated to be particularly advantageous for continuous real-time live-cell imaging and show significant potential for use in a wider range of complex bio-imaging applications. Overall, the cellular imaging performance, ease of utility and the selectivity for lysosomal staining could be judged as excellent in comparison with the most recently developed probes^41^.

To complete the imaging portfolio, we wished to illustrate that the bioresponse of **2** within subcellular compartments could also be visualized at the macroscopic scale of a tumour. This translation to *in vivo* tumour imaging is achievable as shown in Fig. 12 in which tumour can be clearly distinguished, showing a high potential for targeted responsive fluorescence imaging.

In conclusion, the development of **2** as the first phenol/phenolate controlled molecular NIR lysosome-responsive fluorophore has important implications for the study of intracellular transport mechanisms, lysosome-based diseases and *in vivo* targeting. The use of **2** for further 4D real-time studies of more complex dynamic cell mechanisms involving lysosomes are ongoing. Future studies will include the conjugation of active tumour targeting motifs via **7** (instead of PEG) to the fluorophore, to further broaden the possibilities of *in-vivo* imaging targets with potential applications for fluorescence-guided surgery.

## Methods

### General information and materials

All commercially available solvents and reagents were used as supplied, unless otherwise stated. All reactions were performed under nitrogen or argon atmosphere in oven-dried glassware. Gel chromatography was performed with Davisil 60 silica (230–400 mesh). On the basis of NMR and reverse-phase HPLC, all final compounds were >95% pure. ^1^H and ^13^C spectra were recorded on 300, 400, 500 or 600 MHz NMR spectrometers and chemical shifts were reported in p.p.m. using solvent residual peak as standard. For spectra of compounds **4**, **5**, **6** and **7**, see Supplementary Figs 10–13, respectively. All ^19^F NMR chemical shifts are referenced to CFCl_3_. All ^11^B NMR chemical shifts are referenced to BF_3_.Et_2_O/CDCl_3_. High-resolution mass spectrometry and tandem mass spectrometry experiments were carried on an electrospray ionisation and MALDI–TOF instruments. Infrared spectra were recorded as a KBr pellet using a Fourier Transform infrared spectrometer. Absorbance spectra were recorded with a Varian Cary 50 Scan ultraviolet–visible spectrometer. Fluorescence spectra were recorded with a Varian Cary Eclipse Fluorescence Spectrometer. Solvents for absorbance and fluorescence experiments were of HPLC quality. SigmaPlot, MestreNova, ChemDraw, Zeiss LSM and ImageJ software were used for data analysis. Phenol red-free imaging DMEM medium was used for all experiments.

### Synthesis of compound **4**

Compound (ref. 28) (200 mg, 0.38 mmol) and CsF (288 mg, 1.89 mmol) were dissolved in dry DMSO (6 ml) and stirred at 30 °C under a nitrogen atmosphere for 10 min, during which time the colour changed from dark green to dark purple. *t*Butyl bromoacetate (126 mg, 0.64 mmol) was then added via syringe in one go and the solution was stirred at 30 °C for 20 min. The mixture was partitioned between AcOEt (100 ml) and PBS buffer at pH 7 (100 ml). The organic phase was washed with water (3 × 100 ml), brine (50 ml), dried over Na_2_SO_4_, filtered and evaporated to dryness. The crude product was purified by silica gel chromatography, eluting with CH_2_Cl_2_:AcOEt (99:1→90:10) to yield the product **4** as a red metallic solid (151 mg, 64%). mp: 183–187 °C; ^1^H NMR (400 MHz, DMSO-*d*_*6*_): *δ* 10.58 (br s, 1H), 8.19–8.10 (m, 8H), 7.65 (s, 1H), 7.57–7.43 (m, 7H), 7.10 (d, J=8.7 Hz, 2H), 6.95 (d, *J*=8.6 Hz, 2H), 4.81 (s, 2H), 1.46 (s, 9H); ^13^C NMR (100 MHz, DMSO-*d*_*6*_): *δ* 167.5, 161.5, 160.0, 158.9, 155.7, 145.0, 143.8, 142.6, 141.0, 132.5, 132.0, 131.7, 131.4, 129.6, 129.3, 129.1, 129.0, 128.7, 128.6, 124.1, 121.3, 120.3, 119.1, 116.0, 114.8, 81.6, 65.0, 27.7; ^11^B NMR (128 MHz, DMSO-*d*_*6*_) *δ*: 1.00 (t, *J*=32.6 Hz); ^19^F NMR (376 MHz, DMSO-*d*_*6*_): *δ* −130.44 (q, *J*=32.6 Hz); ultraviolet–visible: *λ*_max_ (CHCl_3_): 680 nm (*ɛ*: 85,000 cm^−1^ M^−1^); emission: *λ*_max_ (CHCl_3_): 708 nm, Φ_F_ (CHCl_3_)=0.31; high resolution mass spectrometry (HRMS) (*m*/*z*): [M-H]^−^ calcd. for C_38_H_31_BN_3_O_4_F_2_, 642.2376; found, 642.2357.

### Synthesis of compound **5**

A solution of **4** (300 mg, 0.48 mmol) in acetonitrile (6 ml) was heated under reflux for 5 min. A solution of KNO_3_ (53 mg, 0.53 mmol) and KHSO_4_ (130 mg, 0.96 mmol) in water (1 ml) was added and the mixture was heated under reflux for 5 min. The suspension was cooled to room temperature (rt) and partitioned between AcOEt (200 ml) and water (100 ml). The organic phase was washed with water (100 ml), brine (2 × 100 ml), dried over Na_2_SO_4_, filtered and evaporated to dryness. The crude was purified by silica gel chromatography, eluting with CH_2_Cl_2_:AcOEt (99: 1) to yield the product **5** as a dark red metallic solid (198 mg, 60%). mp: 192–194 °C; ^1^H NMR (400 MHz, DMSO-*d*_*6*_): *δ* 11.88 (br s, 1H), 8.69 (d, *J*=2.0 Hz, 1H), 8.27 (dd, *J*=8.9, 2.0, 1H), 8.21 (d, *J*=9.0 Hz, 2H), 8.19–8.13 (m, 4H), 7.71 (s, 1H), 7.60 (s, 1H), 7.57–7.43 (m, 6H), 7.27 (d, *J*=8.9 Hz, 1H), 7.12 (d, *J*=9.0 Hz, 2H), 4.84 (s, 2H), 1.45 (s, 9H); ^13^C NMR (100 MHz, DMSO-*d*_*6*_): *δ* 167.4, 160.9, 159.3, 154.1, 153.9, 145.4, 143.9, 143.3, 141.5, 137.3, 135.6, 132.1, 131.8, 131.5, 129.9, 129.5, 129.2, 129.0, 128.7, 128.7, 126.6, 123.3, 121.8, 120.8, 119.4, 119.1, 115.0, 81.7, 65.1, 27.7; ^11^B NMR (128 MHz, DMSO-*d*_*6*_): *δ* 0.92 (t, *J*=32.7 Hz); ^19^F NMR (376 MHz, DMSO-*d*_*6*_): *δ* −130.31 (q, *J*=32.7 Hz); ultraviolet–visible: *λ*_max_ (CHCl_3_) 675 nm (*ɛ*: 94,000 cm^−1^M^−1^); emission: *λ*_max_ (CHCl_3_) 703 nm, Φ_F_ (CHCl_3_)=0.15; HRMS (*m*/*z*): [M-H]^−^ calcd. for C_38_H_30_BN_4_O_6_F_2_, 687.2226; found, 687.2229.

### Synthesis of compound **6**

TFA (1 ml) was added dropwise to a solution of **5** (175 mg, 0.25 mmol) in dichloromethane (DCM) (9 ml) and the solution was stirred at rt for 3 h. The solvent was removed under vacuo and the residual TFA was removed azeotropically with serial additions of DCM and subsequent removal under vacuo. The solid was suspended in DCM, filtered and washed with DCM, to yield the product as a dark purple solid (136 mg, 84%). The product was pure enough to proceed to the next synthetic step. To remove the last trace of starting material, the solid was partitioned between AcOEt (90 ml) and Na_2_CO_3_ sat. (180 ml). The organic layer was discarded and the water layer was extracted with AcOEt (90 ml), separated, carefully acidified with 5 M HCl and extracted again with AcOEt (180 ml). The organic layer was separated, dried over anhydrous Na_2_SO_4_, filtered and evaporated to dryness. The product **6** was obtained as a dark purple solid (122 mg, 76%). mp: 214–219 °C; ^1^H NMR (400 MHz, DMSO-*d*_*6*_): *δ* 13.15 (br s, 1H), 8.68 (d, *J*=2.3 Hz, 1H), 8.28 (dd, *J*=8.9, 2.3 Hz, 1H), 8.24–8.13 (m, 6H), 7.71 (s, 1H), 7.60 (s, 1H), 7.57–7.44 (m, 6H), 7.28 (d, *J*=8.9 Hz, 1H), 7.14 (d, *J*=9.0 Hz, 2H), 4.87 (s, 2H); ^13^C NMR (100 MHz, DMSO-*d*_*6*_): *δ* 169.7, 161.0, 159.4, 154.1, 153.8, 145.4, 143.9, 143.4, 141.5, 137.3, 135.5, 132.1, 131.9, 131.5, 129.9, 129.5, 129.2, 129.0, 128.7 (2C), 126.6, 123.2, 121.9, 120.8, 119.5, 119.1, 115.1, 64.6; ^11^B NMR (128 MHz, DMSO-*d*_*6*_): *δ* 0.93 (t, *J*=32.8 Hz); ^19^F NMR (376 MHz, DMSO-*d*_*6*_): *δ* −130.34 (q, *J*=32.8 Hz); HRMS (*m*/*z*): [M-H]^−^ calcd. for C_34_H_22_BN_4_O_6_F_2_, 631.1600; found, 631.1603.

### ^1^H NMR monitoring of the formation of activated ester **6**

A mixture of **5** (45 mg, 0.071 mmol), *N*-(3-dimethylaminopropyl)-*N*′-ethylcarbodiimide hydrochloride (27 mg, 0.14 mmol) and *N*-hydroxysuccinimide (82 mg, 0.71 mmol) was placed in a sealed dry flask. Anhydrous deuterated DMSO-*d*_*6*_ (1.2 ml) was added to the mixture and the solution was stirred at rt under N_2_ atmosphere. Samples (50 μl) were withdrawn at 15, 30, 60, 120 min and 19 h, diluted with DMSO-*d*_*6*_ in an NMR tube (650 μl) and ^1^H spectra were recorded at a 600-MHz spectrometer.

### Synthesis of compound **7**

A mixture of **6** (40 mg, 0.063 mmol), *N*-(3-dimethylaminopropyl)-*N*′-ethylcarbodiimide hydrochloride (24 mg, 0.13 mmol) and *N*-hydroxysuccinimide (73 mg, 0.63 mmol) was dissolved in anhydrous DMSO (1 ml) and stirred at rt for 3 h under N_2_ atmosphere. The solution was partitioned between with DCM (50 ml) and 0.5 M HCl (50 ml). The organic phase was washed with 0.5 M HCl (50 ml), acidic brine (50 ml), dried over Na_2_SO_4_, filtered and evaporated to dryness, keeping the temperature of the bath below 35°C. The product **7** was obtained as a purple metallic solid (44 mg, 95%). m.p.: 177–183 °C; ^1^H NMR (400 MHz, DMSO-*d*_*6*_): *δ* 8.69 (d, *J*=2.2 Hz, 1H), 8.29 (dd, *J*=8.9, 2.2 Hz, 1H), 8.23 (d, *J*=8.9 Hz, 2H), 8.21–8.14 (m, 4H), 7.72 (s, 1H), 7.64 (s, 1H), 7.59–7.45 (m, 6H), 7.28 (d, *J*=8.9 Hz, 1H), 7.23 (d, *J*=8.9 Hz, 2H), 5.53 (s, 2H), 2.85 (s, 4H); ^13^C NMR (100 MHz, DMSO-*d*_*6*_): *δ* 169.9, 165.2, 159.8, 158.6, 154.5, 145.2, 144.2, 143.1, 142.0, 137.4, 135.5, 132.0, 131.8, 131.6, 129.8, 129.6, 129.2, 129.1, 128.7, 126.8, 124.1, 121.5, 120.6, 119.6, 119.5, 115.2, 63.0, 25.5; ^11^B NMR (128 MHz, DMSO-*d*_*6*_): *δ* 0.93 (t, J=32.8 Hz); ^19^F NMR (376 MHz, DMSO-*d*_*6*_): *δ* −130.34 (q, *J*=32.8 Hz); HRMS (*m*/*z*): [M-H]^−^ calcd. for C_38_H_25_BF_2_N_5_O_8_, 728.1764; found, 728.1730.

### Synthesis of compound **2**

A mixture of **7** (6.4 mg, 0.0088, mmol) and *O*-(2-aminoethyl)polyethylene glycol 5000 (CAS 32130–27–1) (40 mg, 0.008 mmol) was dissolved in anhydrous DMSO (0.88 ml) and stirred at rt for 18 h under a N_2_ atmosphere. The solvent was removed by short-path distillation at rt overnight and the crude was partitioned between DCM (20 ml) and 1 M Na_2_CO_3_ (20 ml). The aqueous phase was extracted with DCM (2 × 20 ml). The organic layers were combined, washed with slightly acidic (HCl) water (20 ml), brine (20 ml), dried over anhydrous Na_2_SO_4_, filtered and evaporated to dryness. The residue was dissolved in HPLC grade water (8 ml) and the dark solution was passed through a Sep Pak C18 reverse-phase cartridge, then freeze dried. The product **2** was obtained as a dark green solid (40 mg, 90%). mp: 43–45 °C; ^1^H NMR (400 MHz, DMSO-*d*_*6*_): *δ* 8.74–8.72 (m, 1H), 8.28 (dd, *J*=9.0, 2.3 Hz, 1H), 8.24–8.15 (m, 7H), 7.68 (s, 2H), 7.58–7.52 (m, 4H), 7.52–7.45 (m, 2H), 7.25–7.19 (m, 1H), 7.16 (d, *J*=9.0 Hz, 2H), 4.66 (s, 2H), 3.70–3.65 (m, 4H), 3.50 (s, 680H); Ultraviolet–visible: *λ*_max_ (CHCl_3_) 670 nm, (*ɛ*: 97,000 cm^−1^M^−1^); emission: *λ*_max_ (CHCl_3_) 702 nm, Φ_F_ (CHCl_3_)=0.18; HRMS (m/z): MALDI–TOF distribution maximum centred at 5410.3999 Da.

### Fluorescence quantum yields and extinction coefficients

The compound of interest (0.005 mmol) was dissolved in CHCl_3_ (50 ml) to prepare a stock solution (10^−4^ M). The stock was diluted to concentrations 2, 4, 6, 8 and 10 × 10^−7^ M with CHCl_3_ and each solution was analysed with an ultraviolet–visible spectrometer and a fluorescence spectrometer against CHCl_3_ background. Excitation=640 nm; emission range=660–900 nm; slit width=5/5 nm; scan rates=600 nm min^−1^. Plots of abs_max_ versus conc and fluorescence area versus abs (640 nm) allowed the calculation of extinction coefficient and fluorescence quantum yield, respectively. Compound **1** (R=Ph, Ar=*p*MeOC_6_H_4_) was used as standard for fluorescence quantum yields with Φ_F_=0.36 (refs 17, 42).

### Fluorescence response of **2** and **5** to addition of DBU/TFA in organic solvents

Compound **2** or **5** was dissolved in toluene, tetrahydrofuran, dimethylformamide and DMSO (25 ml) to a final concentration of 5 μM. A solution of DBU (29.5 mg in 100 ml of CHCl_3_) was added (64 μl=1 eq) gradually, and absorbance and fluorescence spectra were recorded before and after each addition. The addition was stopped once spectra remained unchanged. At this stage, an excess of DBU was added and the spectra were recorded. Subsequently, a higher excess of TFA was added and the spectra were recorded. The area below the last two curves was plotted for off/on histogram (shown in Fig. 3). (Note: the toluene solution of **2** contained 1% CHCl_3_ for solubility).

### Fluorescence response of **2** to pH variation in DMEM

Compound **2** (2.8 mg) was dissolved in PBS (500 μl). The stock solution (1 mM) was diluted with DMEM supplemented with 10% FBS to the concentration of 5 μM. The pH of the solution was adjusted with diluted HCl or NaOH, to obtain a range from 8 to 2 at regular intervals, each of which was recorded, and the respective solution analysed by ultraviolet–visible absorption and fluorescence emission. Excitation=625 nm; emission range=635–900 nm.

### Comparative solution and cellular photobleaching of **2** and lysotracker red and pHrodo red

Entire fluorescence cuvettes contain 1 × 10^−7^ M DMEM solutions at pH 4.0 of **2**; lysotracker red and pH-rodo red were continuously irradiated with light of wavelength 620(30) nm for **2** and 540(40) nm for lysotracker red and pH-rhodo red at 25 °C for 2 h. Filtered light from a 150-W light source used with complete cuvette irradiation via a fibre optic with attached light diffuser. Fluorophore fluorescence intensities were recorded every 20 min. The average fluorescence intensity from three independent experiments were normalized and plotted with sigmaplot 8.

Ten thousand HeLa-Kyoyo cells in DMEM were seeded onto chamber slides and incubated with **2** (20 μM) for 60 min or lysotracker red (150 nM) for 30 min, or pH rhodo red (15 μM) for 30 min. DMEM was replaced with fluorophore-free media and cells constantly irradiated with a Lumencor SPECTRA light engine LED used as the light source set to a maximum power for 400 s. Excitation filter 563/9 nm was used for lysotracker red and pH-rhodo red and excitation filter 640(14) nm was used for **2**. Cells were imaged with the shutter open, a time intervals of either 0.1 or 1.0 or 5 s with exposure of 10 ms and individual frames complied into movie format. The average cellular ROI fluorescence intensities from three independent experiments were plotted. An Olympus × 60 PLANAPO/1.42 objective and Andor iXon 888 ultra were used for signal detection. Acquisition and analysis performed with MetaMorph v7.8.

### MTT assay of **2**

Compound **2** (4.0 mg) was dissolved in sterile PBS (71 μl) to prepare a stock solution 10 mM. This was serially diluted to prepare samples at 5, 1, 0.5, 0.1 and 0.05 mM. Each of the stock solutions was diluted 1:10 with DMEM medium, which was co-incubated with HeLa or HEK293 cells at 5,000 cells per well on a 96-well plate for 24 h. The solution was removed and substituted with MTT solution (5 mg ml^−1^ in DMEM). The cells were incubated for 3 h. The medium was removed and the wells were treated with DMSO for 10 min. The absorbance of each well was read with a plate reader at 540 nm.

### Production and validation of HeLa Kyoto cell line stably expressing LAMP1-GFP fusion protein

An expression plasmid encoding the LAMP1-GFP fusion protein was generated via the complete open-reading frame coded by a I.M.A.G.E. Fully Sequenced cDNA Clone (Source BioScience, I.M.A.G.E. ID: 5019745) of the human LAMP1 (GenBank accession number BC021288) was amplified by PCR using primers designed to append an XhoI site upstream of the translation initiation site and to replace the translation termination site by a segment encoding EcoRI site followed by a linker sequence CTCCTC (single-letter nucleotide code). The PCR product was gel purified and cloned in the XhoI–EcoRI sites of a pEGFP-N1 vector (BD Biosciences Clontech). Constructs were verified by DNA sequencing.

For stable transfection, HeLa cells were grown at 37 °C in complete DMEM supplemented with 10% FBS and 1% glutamine, to 30–40% confluency, and subsequently transfected with the LAMP1–GFP-encoding plasmid using FuGENE 6 (Roche) following the manufacturer's instruction. One day later, 0.6 g l^−1^ G418 was added. The medium was changed every day to remove the G418 non-resistant cells and when the cell number looked stabilized the G418 was lowered to 0.5 g l^−1^. Cells displaying resistance to G418 and expressing LAMP1-GFP (as judged by fluorescence microscopy) were cloned by limiting dilution and, sorted on a BD FACSAria flow cytometer (Becton Dickinson). The clones were validated by immunostaining, by western blotting and by two functional assays (lysotracker uptake and dextran uptake).

### Microscopy

**Figure 5:**
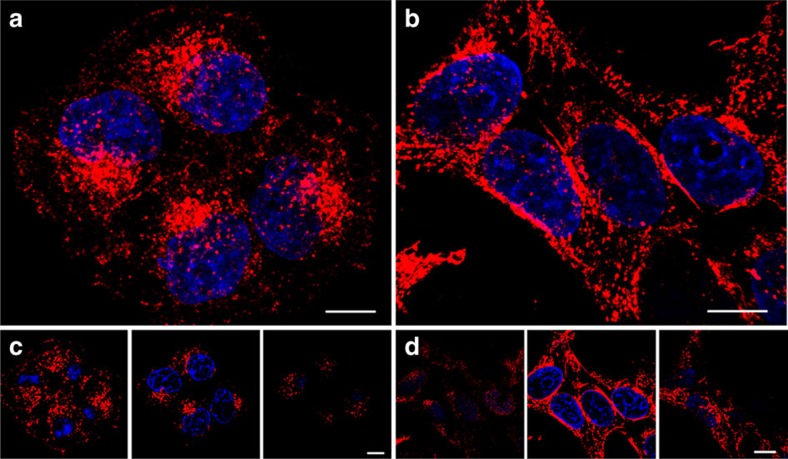
Intracellular NIR-emission profile. CLSM images showing intracellular localization of pH-responsive compound **2** (10 μM, red) and nuclear counterstain Hoechst 33342 (blue) in fixed (**a**,**c**) HeLa Kyoto and (**b**,**d**) HEK293 cell lines. Bottom: three corresponding representative slices of the *Z*-stack for each cell type. Scale bars, 10 μm.

**Figure 6:**
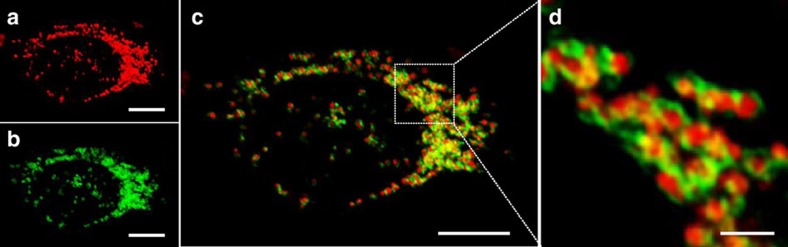
Identification of subcellular NIR-fluorescent on switch. CLSM fluorescent images showing lysosomal localization of the ‘on' state of **2** in LAMP1-GFP-expressing HeLa cells (**a**) Cy5.5 channel; (**b**) GFP channel. (**c**) Three-dimensional image of overlaid Cy5.5 and GFP channels. (**d**) Zoom-in of the dashed box. Scale bars, 10 μm (**a**–**c**) and 2 μm (**d**).

**Figure 7:**
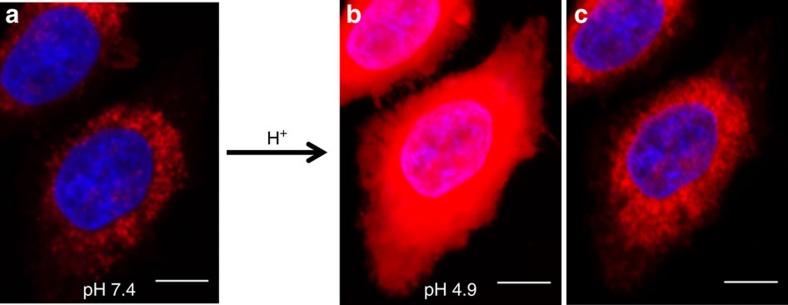
Illustration of NIR-fluorescence response selectivity. (**a**) CLSM imaging of HeLa Kyoto cells following incubation with **2** (10 μM) for 2 h at 37 °C, DAPI nuclei staining and fixing. (**b**) The same set of cells imaged after buffer changed to pH 4.9, keeping the same laser power and PMT voltage. (**c**) The same set of cells after adjustment of microscope laser power and PMT voltage to obtain a non-saturated image. Red: **2**; blue: DAPI stain. Scale bar, 10 μm.

Confocal images (Figs 5, 6, 7) were acquired using an Olympus Fluoview FV1000 CLSM and × 60/1.35(oil) UPLSAPO objective with a 635-nm laser at 12%, PMT voltage of ∼750 v, pixel dwell time of 4 μs per pixel, pixel size 0.103 μm and image size 1,024 × 1,024. Nuclear staining was performed using Hoecscht33342 or DAPI. Hoecshct33342 signal was imaged using a 405-nm laser at 10% power and PMT voltage of ∼700 v. GFP signal was imaged using a 488 nm laser line at 5% power and PMT voltage of∼600v.

**Figure 8:**
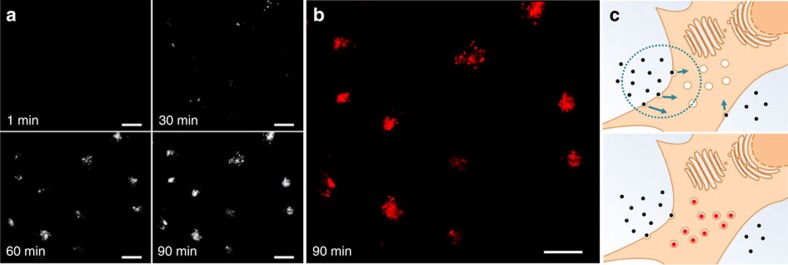
Widefield live-cell imaging of the uptake of 2 (10 μM) into HeLa Kyoto cells. (**a**) Time-lapse black and white images are shown 1, 30, 60 and 90 min. (**b**) Red-coloured image at 90 min. (**c**) Schematic depiction of the uptake process of responsive fluorophore (**c**). Scale bars, 20 nm.

**Figure 9:**
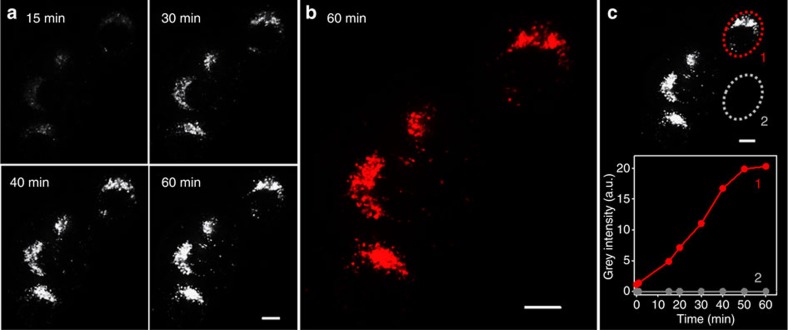
*Z*-axis projections of widefield 4D live-cell imaging of the uptake of 2 (10 μM) from HeLa Kyoto cells. Images were acquired in 25 focal planes every 1 min for 60 min. (**a**) Time lapse b/w images are shown for 15, 30, 40 and 60 min. (**b**) Red-coloured image at 60 min. (**c**) Fluorescence intensity quantification in two identical volumes around a selected cell (1) and in the extracellular environment (2). Scale bars, 10 μm.

**Figure 10:**
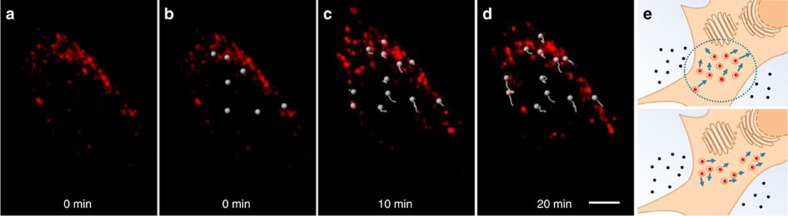
Lysosome tracking in living HeLa Kyoto cells post 1 h incubation with 2 (10 μM). (**a**) Time-lapse representative snapshot of a single cell chosen for image analysis. (**b**) Lysosome selection at 0 min. (**c**,**d**) Tracking over time. (**e**) Schematic depiction of tracking intracellular vesicular movements with bioresponsive fluorophore. Scale bar, 5 μm.

Live-cell images (Figs 8, 9, 10) were acquired on a Zeiss AxioVert 200 M epi-fluorescent widefield microscope equipped with a Andor iXon 885 EMCCD, CoolLED pE-2 solid-state LEDs capable of excitation at 445, 488 and 635 nm, and Zeiss Plan-Apochromat × 100/1.40 Oil DIC objective. The microscope was surrounded by an incubation chamber that allowed the temperature and CO_2_ to be maintained at 37 °C and 5%, respectively. Fluorophore **2** channel was recorded using a 649-nm emission long-pass filter, GFP was imaged using a 520/50 emission bandpass filter.

### Fixed cell imaging

Cells were seeded onto an eight-well chambered glass slide and allowed to attach for 24 h. The media was then replaced with 200 μl of **2** (10 μM) in media and incubated for the appropriate time at 37 °C. Cells were counterstained with Hoechst 33342 or DAPI for 15 min. Cells were then washed once with PBS and fixed in 3.7% paraformaldehyde in PBS solution for 3 min and washed thoroughly with PBS. Images were collected by using an Olympus Fluoview 1,000 CLSM. The fluorescence arising from **2** was detected by a Cy5.5 filter. DAPI and GFP channels were used in parallel when cells were counterstained and/or transfected.

### Fixed cell imaging at different pH

Cells were seeded onto an eight-well chambered glass slide and allowed to attach for 24 h. The media was then replaced with 200 μl of **2** (10 μM) in media incubated for 2 h at 37 °C. Cells were counterstained with DAPI for 15 min. Cells were then washed once with PBS and fixed in 3.7% paraformaldehyde in PBS solution for 3 min, and washed thoroughly with PBS. A collection of cell were *Z*-stack imaged using CLSM (PMT voltage=782v, laser power 12%) and while maintaining focus of the microscope on the same cells the medium was exchanged with medium acidified to pH 4.9 (by addition of HCl (aq)). After allowing 15 min for equilibration the same cells were re-imaged (PMT voltage=782v) using the same laser power. Following which the same cells were imaged for the third time following the adjustment of the PMT voltage 512v to obtain a non-saturated image. Mean total cell fluorescence was determined from two independent experiments using ImageJ.

### Imaging following 4 °C incubation

Cells seeded onto an 8-well chambered glass slide and allowed to attach for 24 h. The media was then replaced with 200 μl of **2** (10 μM) in media incubated for 30 min at 4 °C. Cells were counterstained with DAPI for 15 min. Cells were then washed once with PBS and fixed in 3.7% paraformaldehyde in PBS solution for 3 min and washed thoroughly with PBS. A collection of cell were imaged using CLSM, and while maintaining focus of the microscope on the same cells the medium was exchanged with medium acidified to pH 4.9 (by addition of HCl (aq)). After allowing 15 min for equilibration, the same cells were re-imaged using the same exposure times and laser power.

### Real-time live-cell imaging

HeLa Kyoto cells in Dulbecco's cell growth media containing 10% FBS were seeded onto an eight-well chambered glass slide and incubated for 24 h. The slides were placed on the microscope platform and the microscope was focused on a collection of cells. Next, **2** (final concentration 10 μM) was added and fluorescence images (Cy5.5 filter) were acquired at regular intervals. Images were deconvolved and combined in a video format.

### Time-dependant efflux of **2**

Cells seeded onto an eight8-well chambered glass slide and allowed to attach for 24 h. The media was then replaced with 200 μl of **2** (10 μM) in media incubated for 2 h at 37 °C. Media was replaced with fresh media and the loss of fluorescence monitored over time. Lysosome counting was carried out at 1, 15, 30 and 120 min using ImageJ.

### Image processing

Deconvolution of widefield data sets was performed using AutQuant X3 deconvolution software with ten iterations of adaptive point spread function calculations. Lysosome detection and tracking were performed using Imaris 7.7.1 software (Bitplane Scientific). Background subtraction was applied to all images before lysosome detection. The Spots module of Imaris was used to detect lysosomes with an estimated diameter of 1.27 μm. Detected spots were filtered using the ‘quality' algorithm. Only spots with values higher than the set threshold value (>91.76) were analysed. Quality is defined as the intensity at the centre of the spot, Gaussian filtered by the spot radius. The success and accuracy of Spot detection was judged by visual inspection. Tracking lysosome movement over the course of the video was performed using an autoregressive motion algorithm. A maximum search distance of 1 μm was defined to disallow connections between a spot and a candidate match if the distance between the predicted future position of the spot and the candidate position exceeded the maximum distance. A gap-closing algorithm was also implemented to link track segment ends to track segment starts, to recover tracks that were interrupted by the temporary disappearance of particles. The maximum permissible gap length was set equal to three frames. Tracking all the lysosomes in the cell were selected by applying filters, which were based on ‘Track Length' (>0.2 μm) and ‘Track Duration' (>60 s).

### Statistical analysis of cell images

Manders' and Pearson's coefficients used to show co-compartmentalization of LAMP1–GFP and **2** emissions were calculated using the Image J plugin ‘Coloc2'. Rolling ball background subtraction (50 pixel diameter) and a Gaussian Filter (1 pixel diameter) were applied to all images before running the ‘Coloc2' plugin. The ROI surrounding the cell was selected manually using the freeform drawing tool. Analysis was performed on six cells from two independent experiments.

Corrected total cell fluorescence (CTCF) in Fig. 7 was performed on six cells from two different experiments (Supplementary Fig. 8). *Z*-stack data acquired on the Olympus FLuoview100 was compressed into a single plane using the ‘Sum Slice' function in Image J. Individual cells were selected using the freeform drawing tool to create a ROI (ROI). Selecting the ‘Measure' function provided the area, the mean grey value and integrated density of the ROI. The mean background level was obtained by measuring the intensity in three different regions outside the cells and averaging the values obtained. The CTCF for each cell was calculated using the formula: CTCF=Integrated density of cell ROI−(Area of ROI × Mean fluorescence of background). The FEF was calculated by dividing the CTCF value of a cell at pH 7 into the CTCF value of the same cell at pH 4.9.

**Figure 11:**
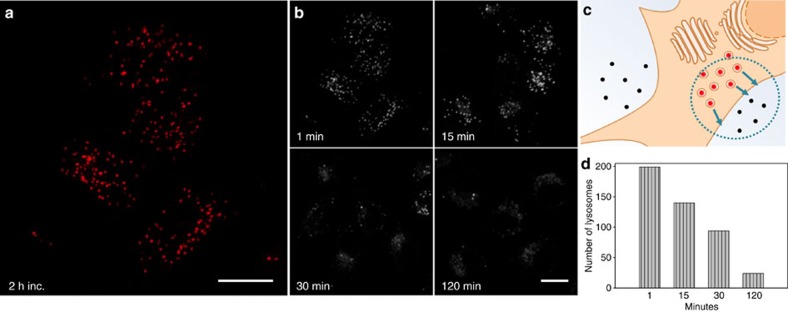
Imaging of cellular efflux. HeLa Kyoto cells were pre-treated with **2** (10 μM) for 2 h, DMEM replaced with fluorophore-free DMEM and cells fixed at various time points. (**a**) Cells imaged after 2 h incubation. (**b**) Cells imaged after 1, 15, 30 and 120 min post media change. (**c**) Schematic depiction of efflux of bioresponsive fluorophore. (**d**) Decrease in number of NIR fluorescent lysosomes from 1 to 120 min. Scale bars, 20 μm.

**Figure 12:**
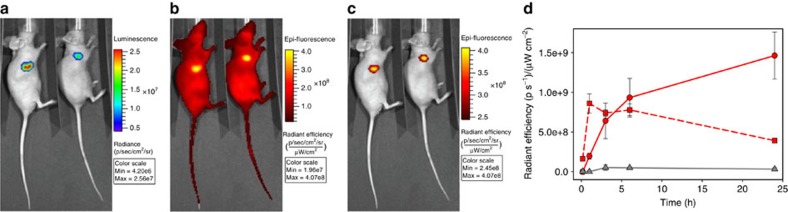
*In vivo* imaging of **2** using a MDA-MB-231-luc-D3H1 subcutaneous tumour model in two representative mice. (**a**) Bioluminescence imaging confirmation of tumour cells. (**b**) NIR fluorescence imaging 24 h post intravenous (i.v.) administration of **2** (excit. 660–690 nm, emis. 710–730 nm). (**c**) NIR fluorescence imaging 24 h post i.v. administration of **2** with intensity scale adjusted (excit. at 675 nm, emiss. at 720 nm). (**d**) Profile of tumour NIR fluorescence (red solid line) and liver (red dashed line) over time following i.v. tail injection of **2**. Non-injected control tumour NIR fluorescence (grey solid line). Values determined from the same sized ROI from background area and tumour averaged for *n*=3.

The number of lysosomes per field of view (Fig. 11) after efflux was counted using *Z*-stack data acquired on the Olympus Fluoview100, which was compressed into a single plane using the ‘Sum Slice' function in Image J. A Max Entropy Threshold 15,000–40,000 was applied to each slice followed by use of the ‘Despeckle', ‘Erode' and ‘Dilate' functions to remove noise. To count the number of lysosomes in each image the ‘Analyze Particles' function was used to count objects with a circularity of 0.75–1.00 and size from 0 to 200 pixels.

### *In vivo* mouse imaging

MDA-MB-231-luc-D3H1, a luciferase-expressing human breast adenocarcinoma cell line, was obtained from Caliper Life Sciences. Cells were maintained as a monolayer culture in minimum essential medium containing 10% (v/v) FBS and supplemented with 1% (v/v) L-glutamine, 50 U ml^−1^ penicillin, 50 μl ml^−1^ streptomycin, 1% (v/v) sodium pyruvate and 1% (v/v) non-essential amino acids. All cells were maintained in 5% CO_2_ (v/v) and 21% O_2_ (v/v) at 37 °C. Balb/C nu/nu mice (Harlan) were housed in the Biomedical Facility (UCD) in individually ventilated cages in temperature and humidity controlled rooms with a 12-h light–dark cycle. Two to five million MDA-MB-231-luc-D3H2LN cells in 100 μl of a DPBS:Matrigel (50:50) solution were injected subcutaneously behind the fore limb of the 5-week-old mice using a 25-g needle. Tumours reached an average diameter of 6 mm before injection. All animal protocols were approved by University College Dublin's local Animal Research Ethics Committee and under the licence from the Department of Health and Children. Animals were split into two groups (*n*=4) and **2** dissolved in PBS (200 μl) was administered through the lateral tail vein at a concentration of 2 mg kg^−1^. Optical imaging was performed with an IVIS Spectrum small-animal *in-vivo* imaging system (Caliper LS) with integrated isoflurane anaesthesia. A non-injected control animal was included. Images were acquired at regular intervals post injection of **2** with excitation 675 nm (30 nm band-pass filter) and emission 720 nm (20 nm band-pass filter) narrow band-pass filters and were analysed using Living Image Software v3.0 (Caliper LS).

## Additional information

**How to cite this article:** Grossi, M. *et al*. Lysosome triggered near infra red fluorescence imaging of cellular trafficking processes in real time. *Nat. Commun.* 7:10855 doi: 10.1038/ncomms10855 (2016).

## Supplementary Material

Supplementary Information Supplementary Figures 1-13 and Supplementary Methods

Supplementary Movie 1 In vitro photobleaching movies of 2.

Supplementary Movie 2 In vitro photobleaching movies of lysotracker red.

Supplementary Movie 3 In vitro photobleaching movies of pH-rhodo red.

Supplementary Movie 4 3D View of Fig. 5.

Supplementary Movie 5 Z-stack of Fig. 6.

Supplementary Movie 6 Z-stack movies of Fig. 7a.

Supplementary Movie 7 Z-stack movies of Fig. 7b.

Supplementary Movie 8 Z-stack movies of Fig. 7c.

Supplementary Movie 9 Movie of Fig. 8.

Supplementary Movie 10 Movie of Fig. 9.

Supplementary Movie 11 Movie of Fig. 10.

Supplementary Movie 12 Movie tracking all lysosomes within the cell shown in Fig. 10.
