# Supplementary material for: Lysosome triggered near-infrared fluorescence imaging of cellular trafficking processes in real time
Source: Nat Commun. 2016 Mar 1;7:10855. doi: 10.1038/ncomms10855 (PMC4773516; doi:10.1038/ncomms10855)
Supplement: Supplementary Information — Supplementary Figures 1-13 and Supplementary Methods [file ncomms10855-s1.pdf]

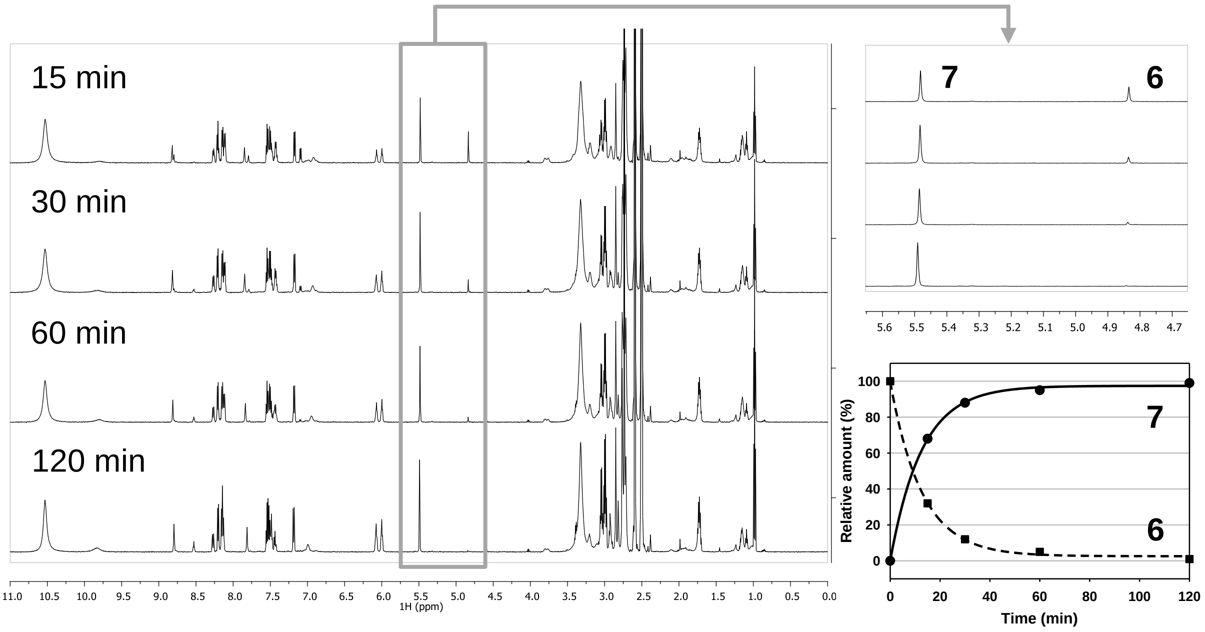

Supplementary Figure 1. ^{1}H NMR Analysis of the Conversion of 6 into 7. Conversion of **6** into **7** monitored by ^{1}H NMR at 15, 30, 60 and 120 min. Diagnostic peaks at 4.83 and 5.48 ppm expanded (top) representing the acid **6** and the active ester **7** respectively; relative integrated area is plotted vs time (bottom) to show nearly full conversion is obtained after 2 h at rt.

(a)

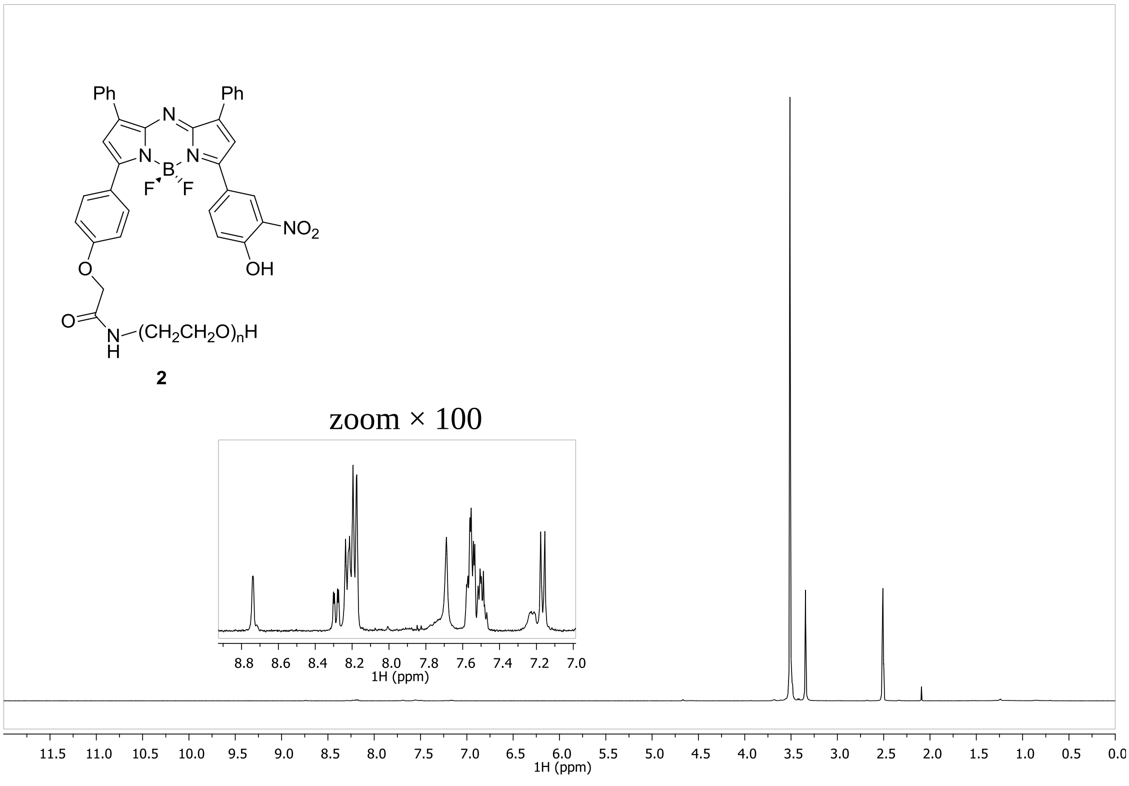
Ph
Ph
N
N
B
N
F
F
NO_2
OH
O
O
N
H
$(CH_2CH_2O)_nH$
2
zoom × 100
1H (ppm)
1H (ppm)

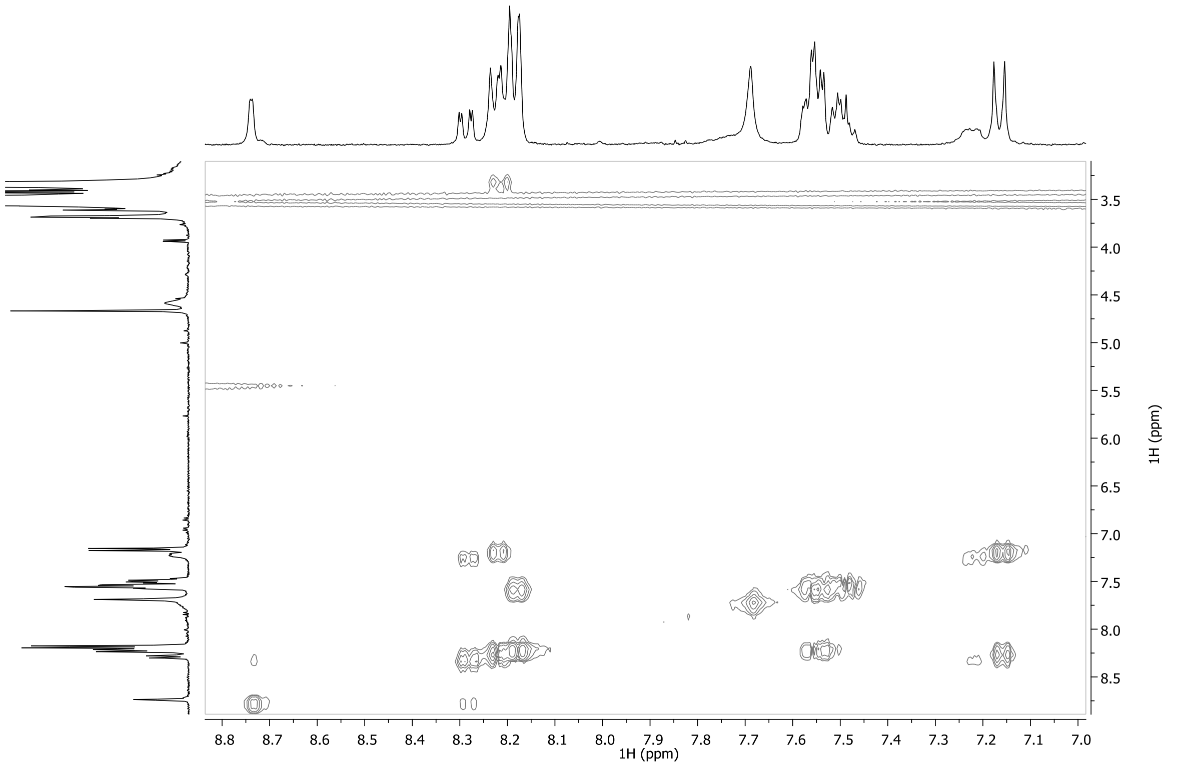
1H (ppm)
1H (ppm)

(**b**)

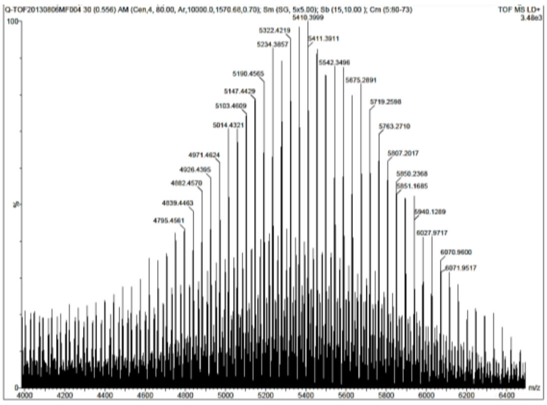

(**c**)

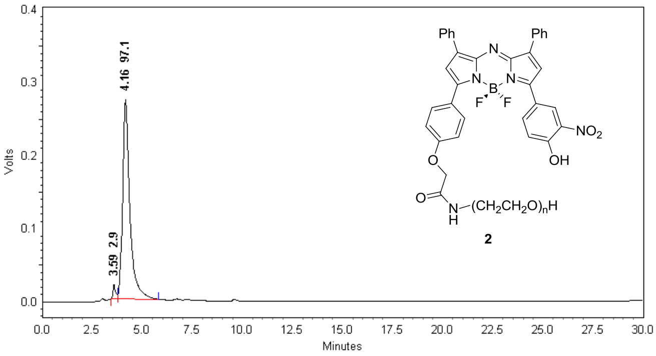

(**d**)

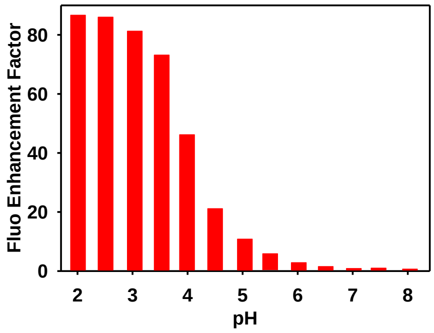

Supplementary Figure 2. Analysis of Fluorophore 2. (**a**) ^{1}H and Cosy Spectra of BF_2 chelate of 2-(4-(5-((5-(4-hydroxy-3-nitrophenyl)-3-phenyl-2*H*-pyrrol-2-ylidene)amino)-4-phenyl-1*H*-pyrrol-2-yl)phenoxy)-*N*-(polyethyleneglycolyl) acetamide **2.** (**b**) MALDI-TOF MS Analyses of **2**. (**c**) HPLC chromatograms of **2**. Conditions: Microsorb-MV 100-5 C18 250 x 4.6 mm reverse phase column; eluent: acetonitrile:water 60:40 containing NH_4HCO_3 10 mM; flow: 0.6 mL/min, UV-detector: 254 nm. (**d**) pH Responsive Fluorescence Enhancement Factor Graph of **2** in DMEM, FEF vs pH graph of **2** (5×10^{-6} M) in DMEM (10% FBS) at pH ranging from 8 to 2 using $FEF_{(pH\ 7.4)}$ = 1. Exc: 625 nm; Cy5.5 filter parameters (690/50 nm) applied to graph in Fig. 4.

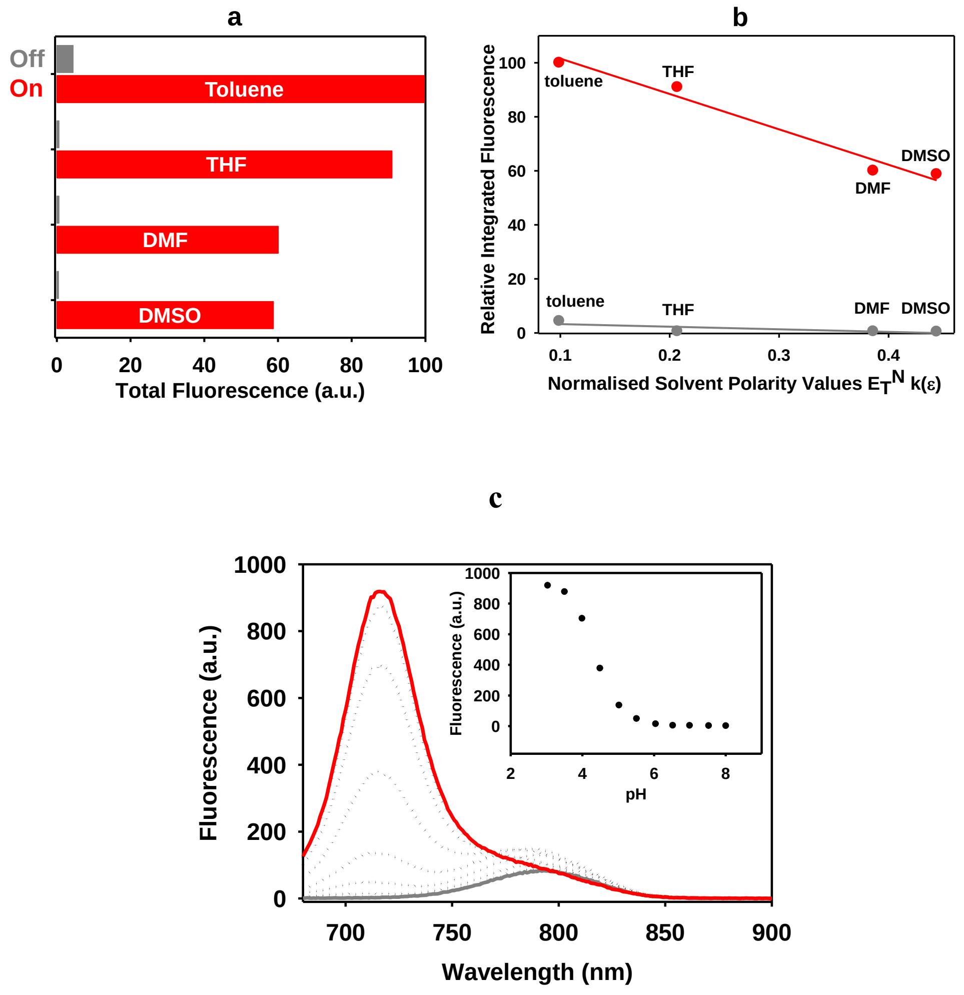

Supplementary Figure 3. Fluorescence switching and polarity response for **5**. (**a**) Integrated off and on fluorescence states of **5** (5×10^{-6} M) in toluene, THF, DMF, and DMSO with TFA (red bars) and DBU (grey bars). (**b**) Plot of relative off and on integrated fluorescence versus solvent polarity values for toluene, THF, DMF and DMSO. **(c)** Fluorescence spectra of **5** (3×10^{-6} M) in CrEL/PBS buffer at pH ranging from 8 (grey) to 3 (red). Exc: 660 nm. Inset: fluorescence intensity at λ_{max} = 716 nm vs pH; sigmoidal plot fit resulted in apparent pK_a = 4.4.

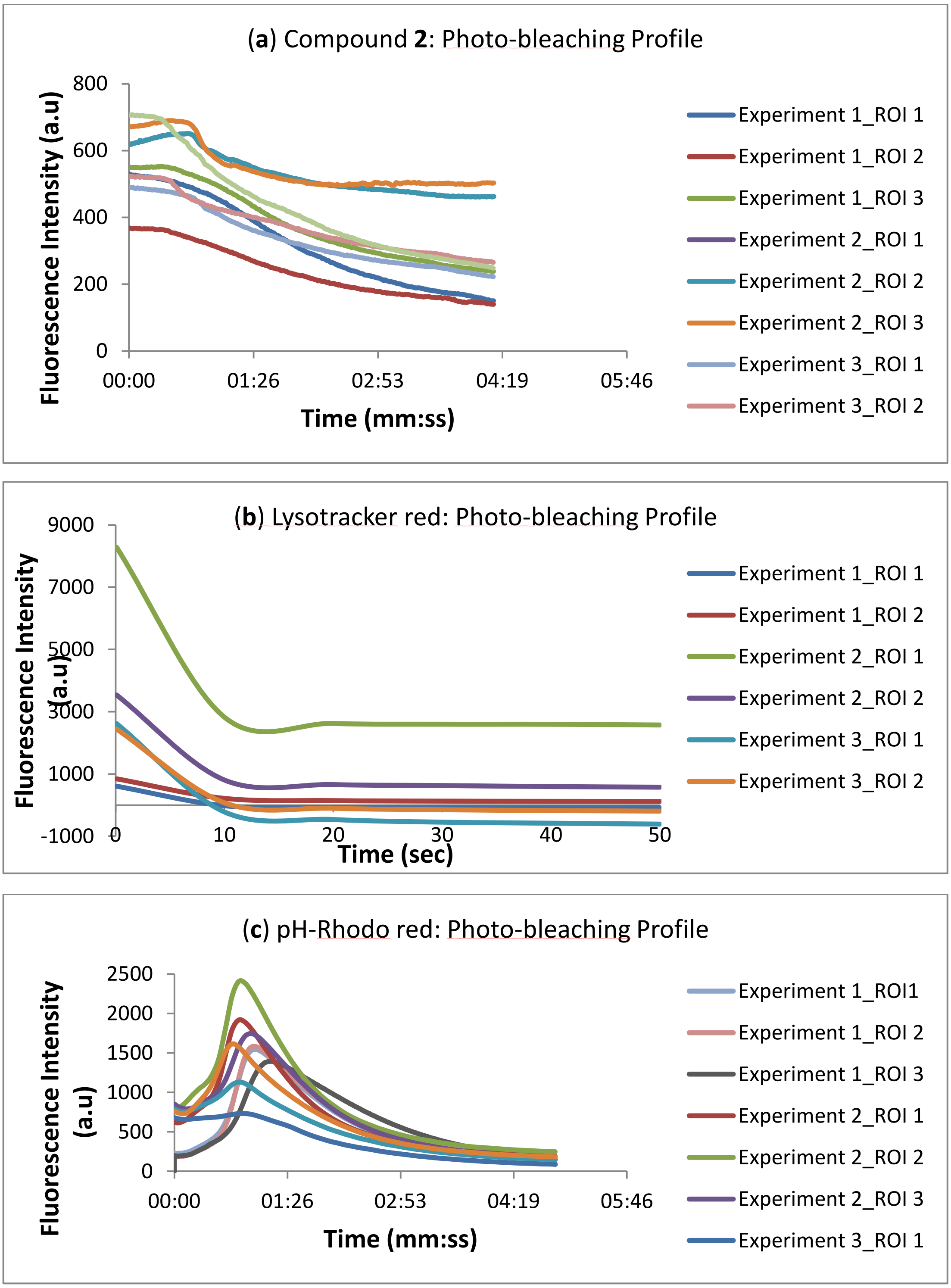

Supplementary Figure 4. Cellular photobleaching profiles for 2, lysotracker red and pH-rhodo red. *In vitro* photobleaching profiles of **2** (**a**), lysotracker red (**b**) and pHrodo red (**c**) with maximum LED power using excitation filter 640(14) nm for **2** and excitation filter 563(9) nm for lysotracker red and pH-rhodo red.

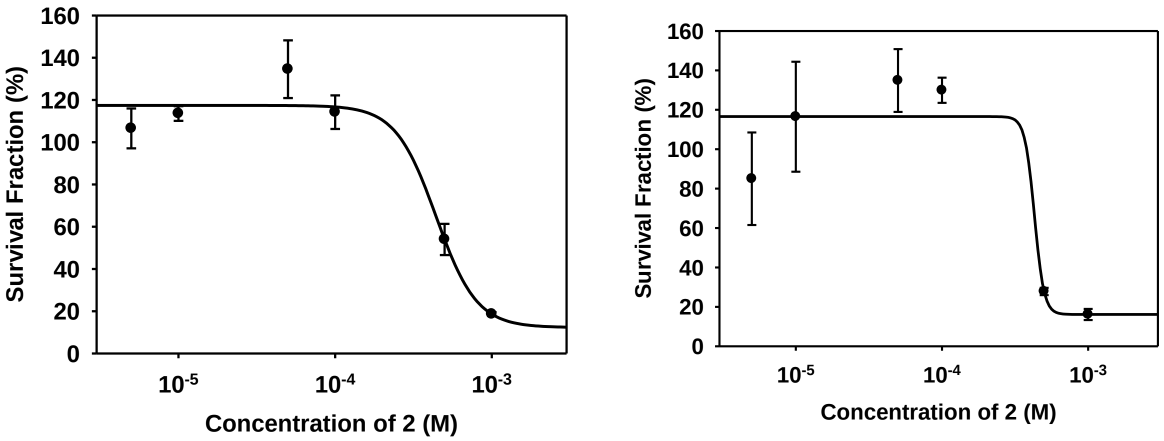

Supplementary Figure 5. MTT Assay of 2 on HeLa Kyoto and HEK293 Cells. MTT colorimetric assay graphs of **2** on HeLa Kyoto (left) and HEK293 (right). IC_{50} values of 0.429 mM (R^2 = 0.8884) and 0.441 mM (R^2 = 0.9555) respectively. Values represent the average of three independent experiments repeated in triplicates.

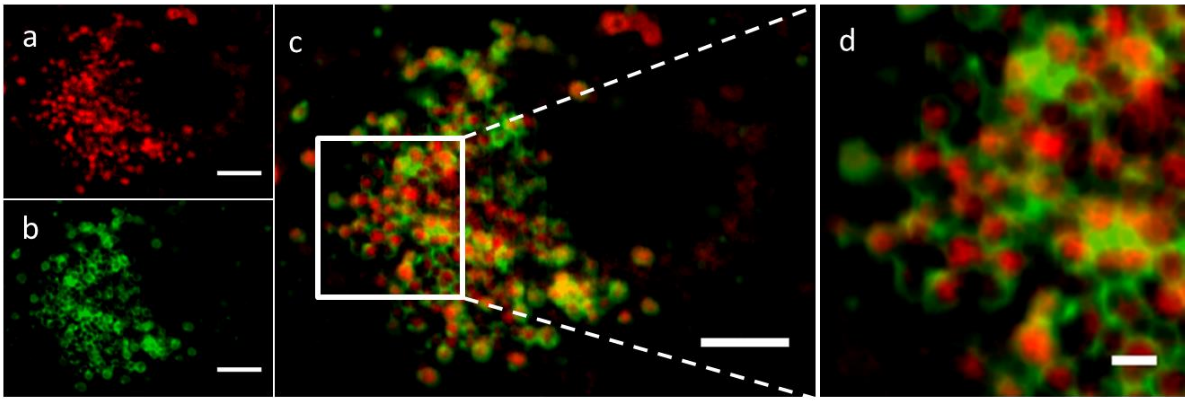

Scale bar, 5µm. Scale bar, 1µm.

Supplementary Figure 6. Additional CLSM fluorescent images showing lysosomal co-compartmentalization of the NIR emission of 2 and LAMP1-GFP in HeLa cells. (**a**) Cy5.5 channel; (**b**), GFP channel. 3D image of overlaid Cy5.5 and GFP channels (**c**) Zoom-in and single representative optical slice of the dashed box (**d**).

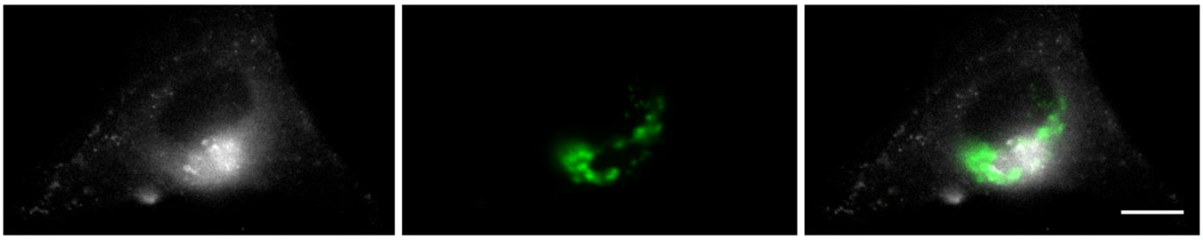

Supplementary Figure 7. HeLa cell golgi (green) co-staining with 2 (white) (scale bar 20 μm).

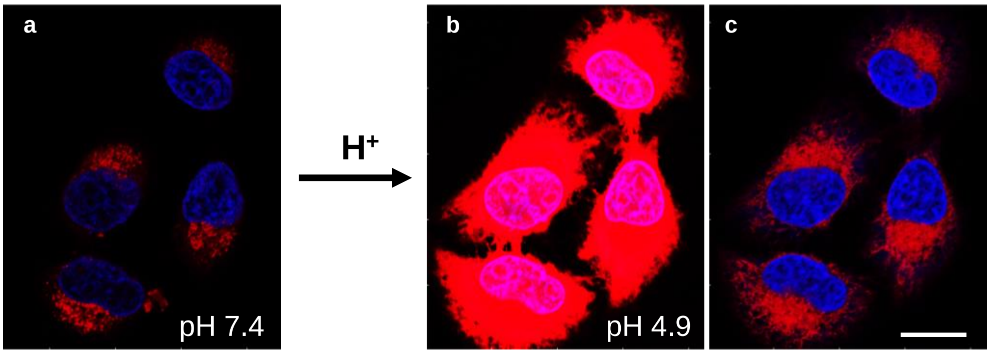

Supplementary Figure 8. Additional CLSM fluorescent images showing pH adjusted cell images. (**a**) CLSM imaging of HeLa Kyoto cells following incubation with **2** (10 µM) for 2 h at 37°C, DAPI nuclei staining and fixing. (**b**) Shows the same set of cells imaged after buffer changed to pH 4.9 keeping the same laser power and exposure time. (**c**) Same set of cells after adjustment of microscope laser power and exposure time to obtain a non-saturated image. Red: **2**; blue: DAPI stain. Scale bar 20 µm.

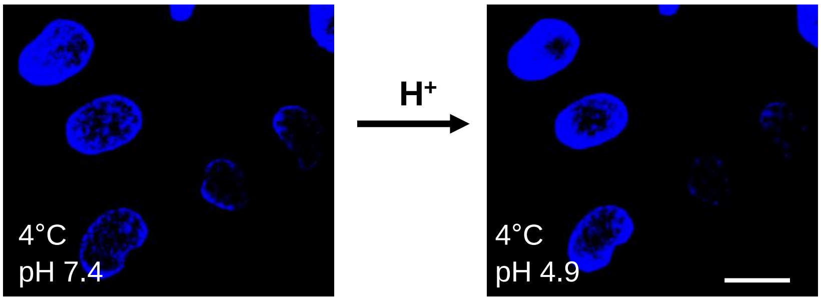

Supplementary Figure 9. CLSM imaging of HeLa Kyoto cells treated with 2 at 4°C. CLSM imaging of HeLa Kyoto cells treated with **2** (10 µM) for 30 min at 4°C. Cells were imaged after fixation and nuclei staining in buffer of pH 7.4 and 4.9. Scale bars 20µm.

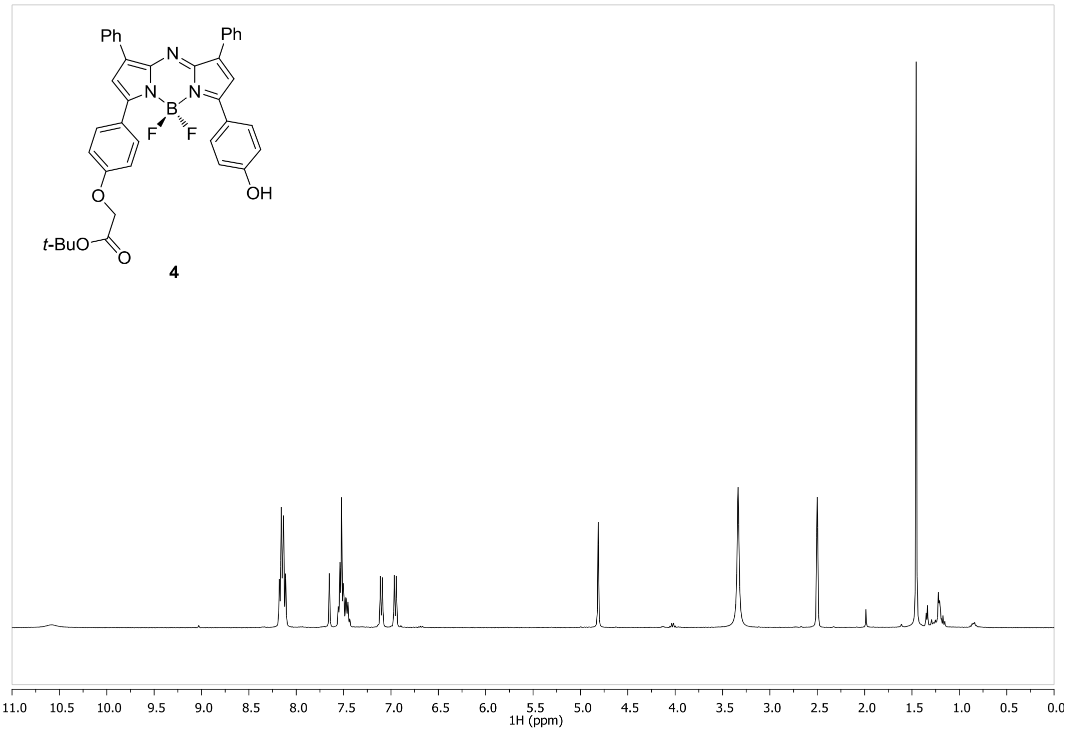

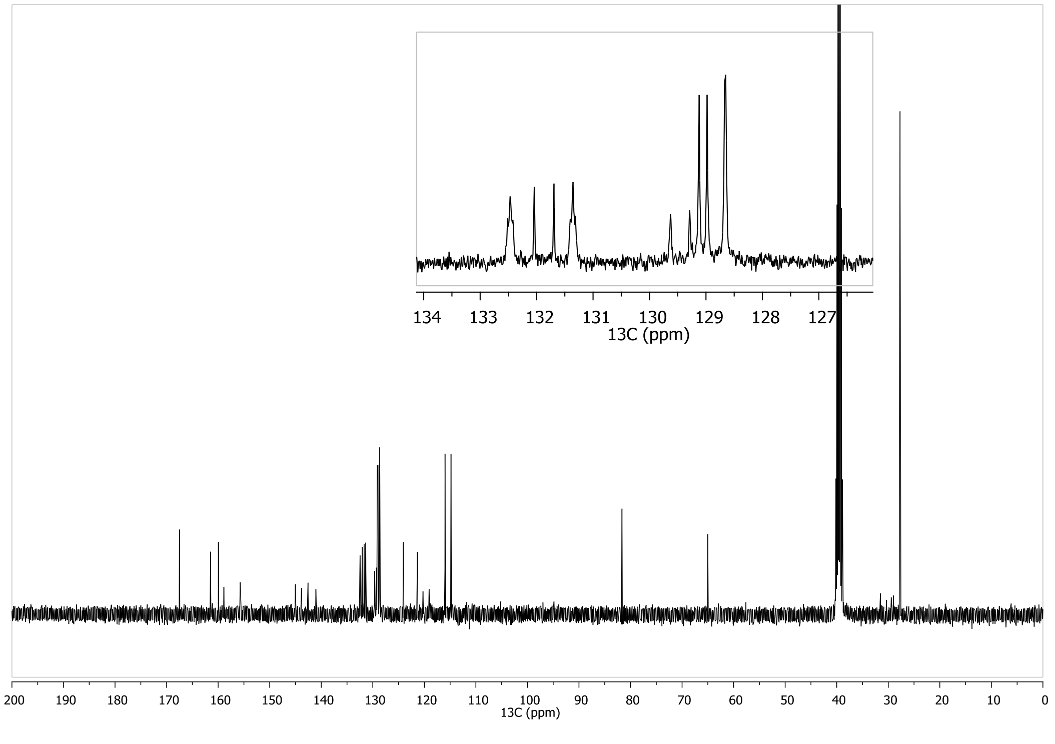

Supplementary Figure 10. ^{1}H and ^{13}C NMR spectra of 4.

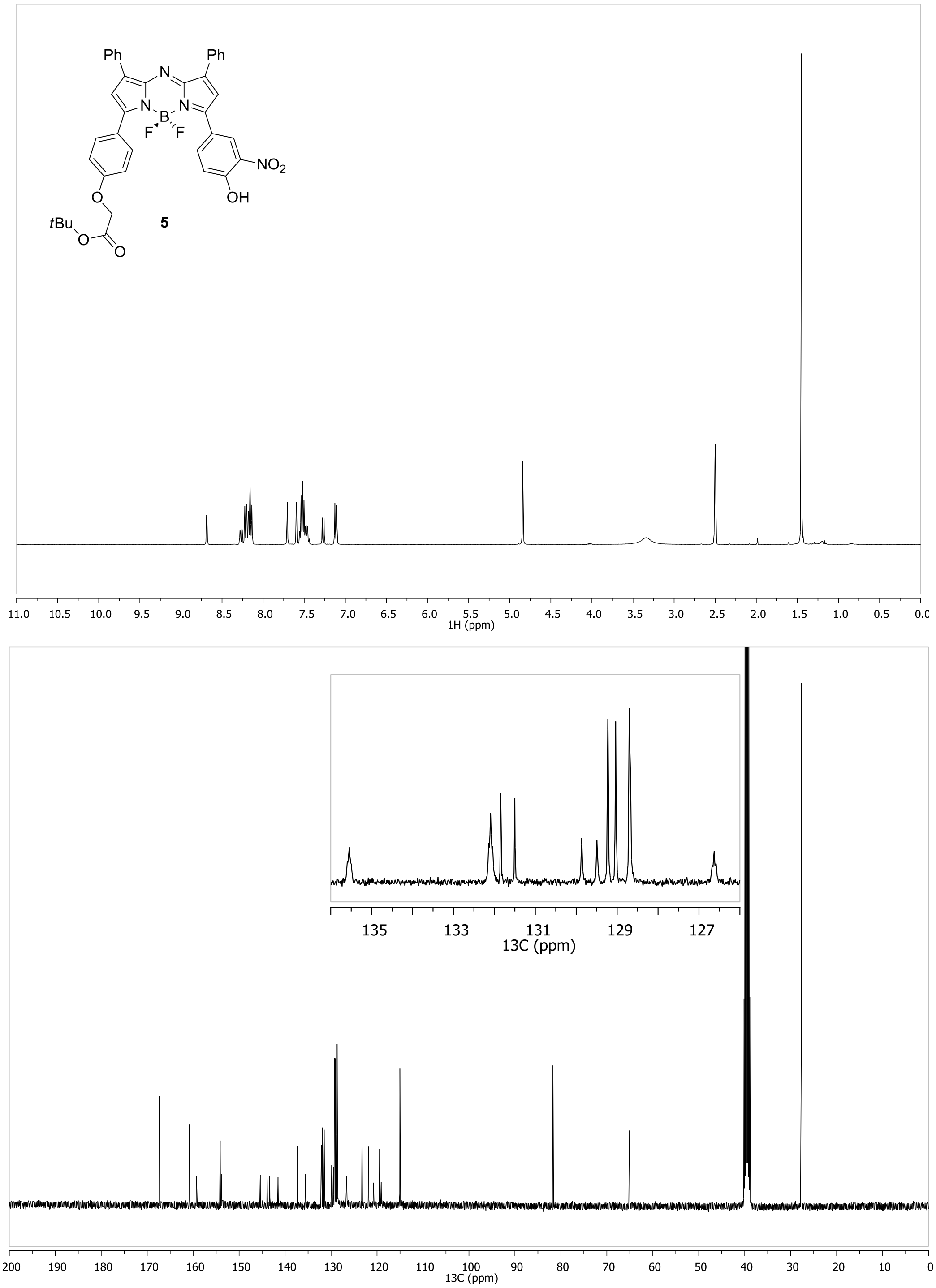

Supplementary Figure 11. ^{1}H and ^{13}C NMR spectra of 5.

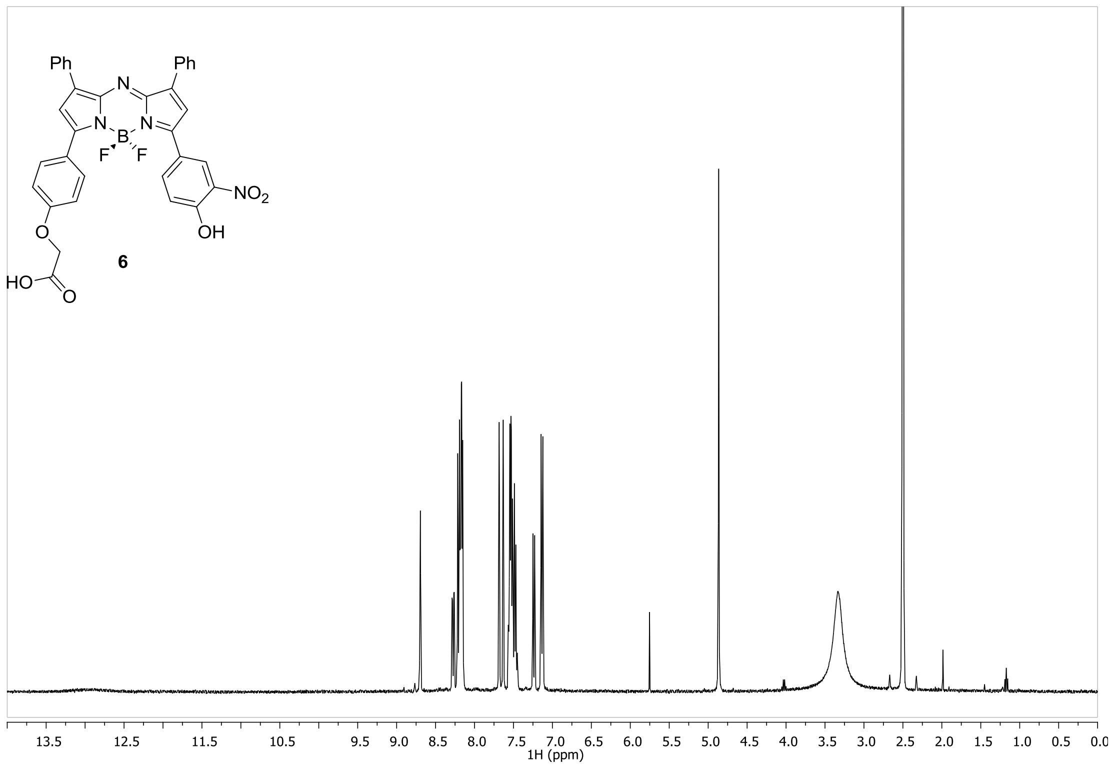
Ph
Ph
N
N
N
B
F
F
NO2
OH
O
HO
O
6
13.5 12.5 11.5 10.5 9.5 9.0 8.5 8.0 7.5 7.0 6.5 6.0 5.5 5.0 4.5 4.0 3.5 3.0 2.5 2.0 1.5 1.0 0.5 0.0
1H (ppm)

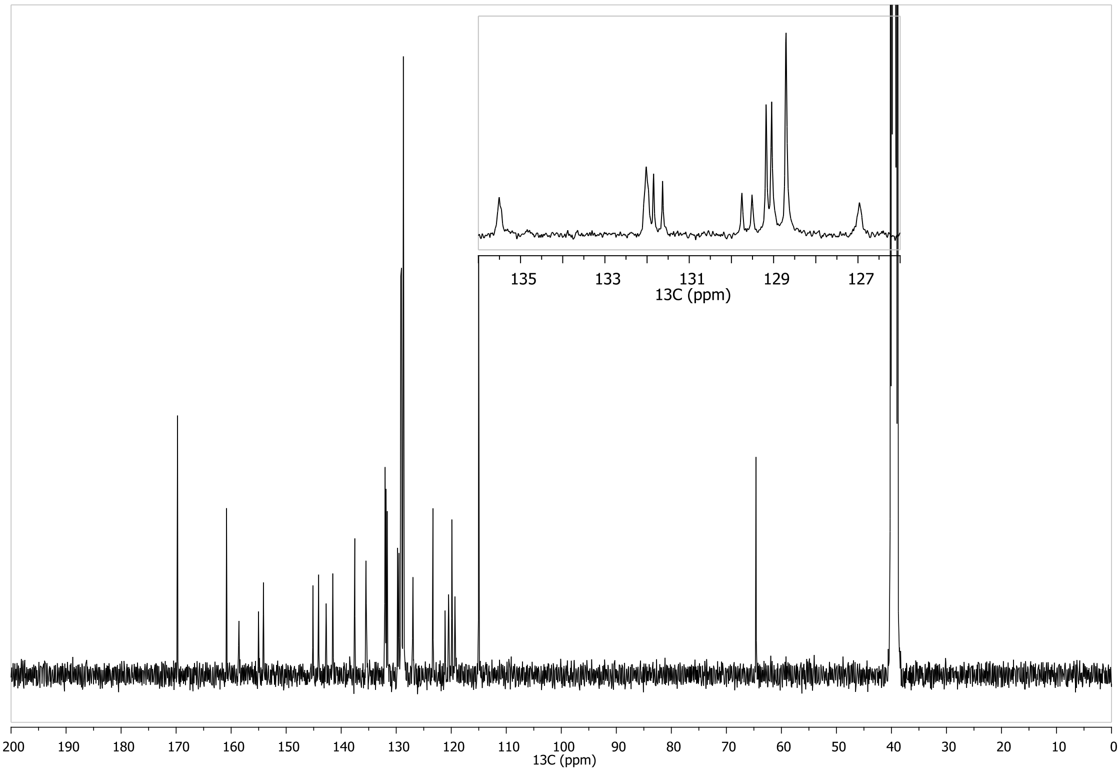
135 133 131 129 127
13C (ppm)
200 190 180 170 160 150 140 130 120 110 100 90 80 70 60 50 40 30 20 10 0
13C (ppm)

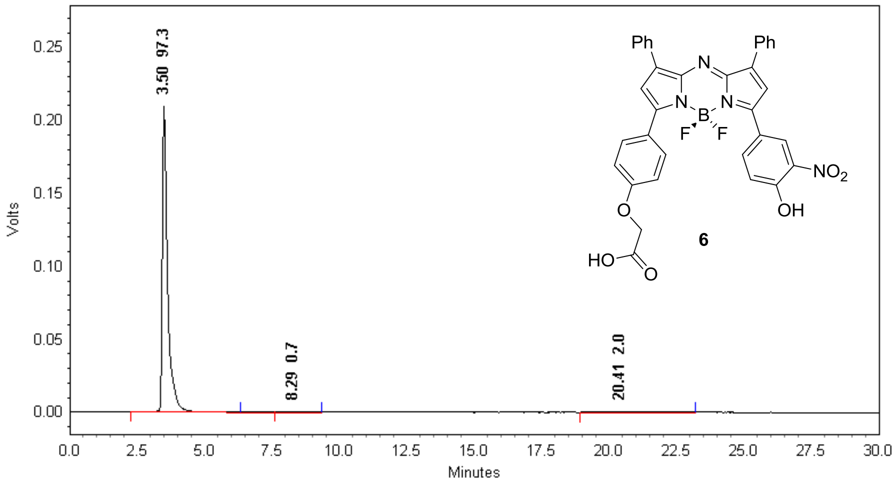

Supplementary Figure 12. ^{1}**H,** ^{13}**C NMR spectra and HPLC of 6.** HPLC chromatograms of **6**. Conditions: Microsorb-MV 100-5 C18 250 x 4.6 mm reverse phase column; eluent: acetonitrile:water 60:40 containing NH_4HCO_3 10 mM; flow: 0.6 mL/min, UV-detector: 254 nm.

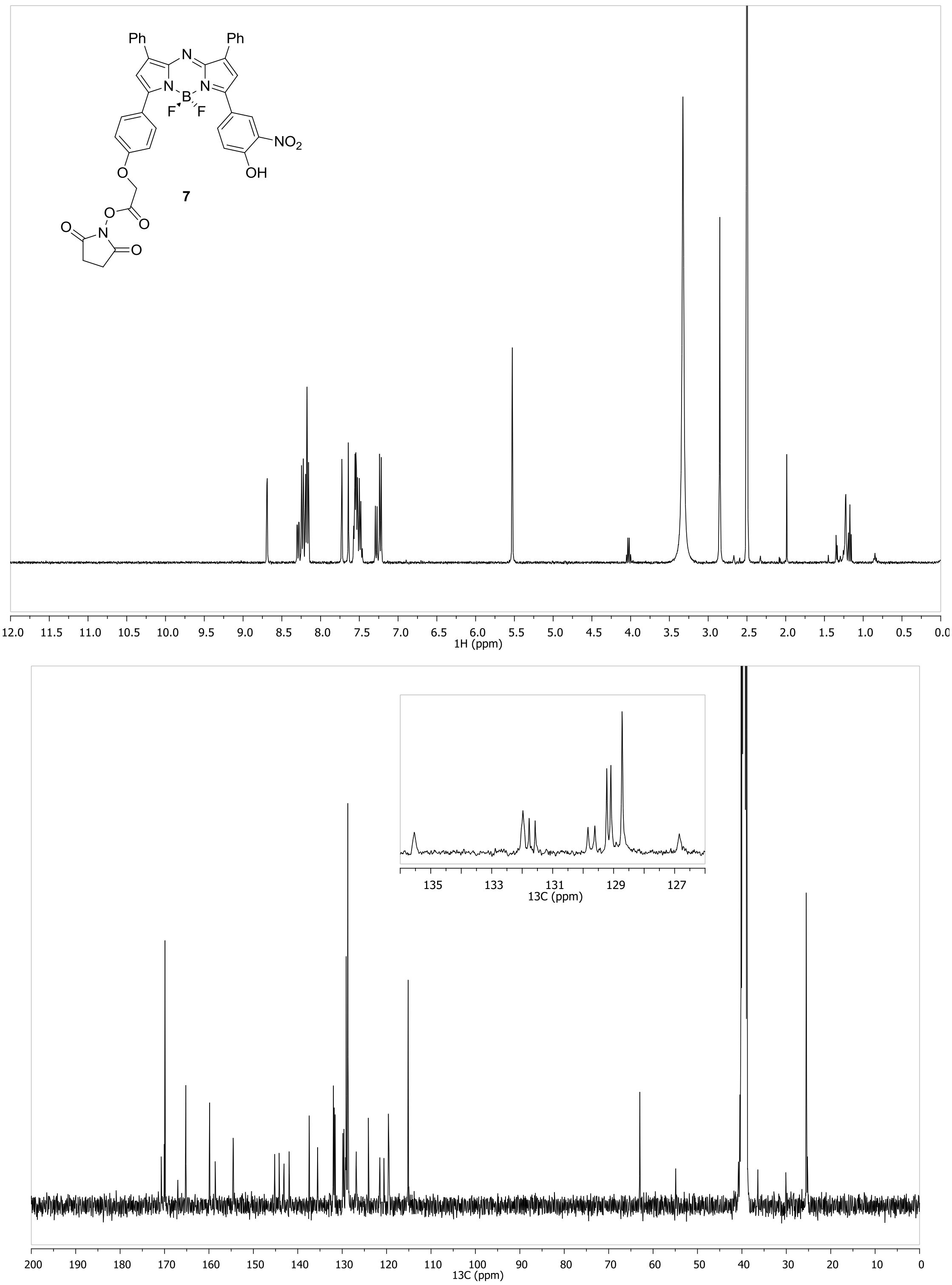

Supplementary Figure 13. ^{1}H **and** ^{13}C **NMR spectra of 7.**

Supplementary Methods. Golgi co-stain with 2.

HeLa – Kyoto were seeded in an eight well Ibidi chamber slide at a density of 10,000 cells per well, allowed to attach overnight and then incubated with **2** for 120 min before fixing with 4% PFA for 20mins. PFA was quenched with 30 mM glycine in PBS / pH 7.5 for 5 min and the cells were permeabilised with 0.1% Triton-X in PBS/ pH 7.5 for 5min. Cells were immersed in monoclonal anti-GM130 antibody (*cis*-Golgi marker) diluted to 1:500 in PBS/ pH7.5 for 45mins, followed by a secondary antibody conjugated to Alexafluor 488 diluted to 1:500 in PBS/ pH7.5 for 45mins. Cells were imaged on an Olympus IX73 microscope using an Olympus 60x 1.45.NA objective.
